# Large-scale intact glycopeptide identification by Mascot database search

**DOI:** 10.1038/s41598-018-20331-2

**Published:** 2018-02-01

**Authors:** Ravi Chand Bollineni, Christian Jeffrey Koehler, Randi Elin Gislefoss, Jan Haug Anonsen, Bernd Thiede

**Affiliations:** 1 Department of Biosciences, University of Oslo, Oslo, Norway; 2 Cancer Registry of Norway, Institute of Population-based Cancer Research, Oslo, Norway

## Abstract

Workflows capable of determining glycopeptides in large-scale are missing in the field of glycoproteomics. We present an approach for automated annotation of intact glycopeptide mass spectra. The steps in adopting the Mascot search engine for intact glycopeptide analysis included: (i) assigning one letter codes for monosaccharides, (ii) linearizing glycan sequences and (iii) preparing custom glycoprotein databases. Automated annotation of both N- and O-linked glycopeptides was proven using standard glycoproteins. In a large-scale study, a total of 257 glycoproteins containing 970 unique glycosylation sites and 3447 non-redundant N-linked glycopeptide variants were identified in 24 serum samples. Thus, a single tool was developed that collectively allows the (i) elucidation of N- and O-linked glycopeptide spectra, (ii) matching glycopeptides to known protein sequences, and (iii) high-throughput, batch-wise analysis of large-scale glycoproteomics data sets.

## Introduction

Protein glycosylation is one of the most common and highly heterogeneous posttranslational modification^1^. More than 50% of the eukaryotic proteins are predicted to be glycosylated and can influence a variety of cellular process^2–6^. Especially, several studies indicated that abnormal glycosylation is strongly associated with many diseases including cancer^7,8^ and many of the FDA approved protein therapeutics and biomarkers are glycoproteins^9–11^.

Many different analytical and MS-based methods have been proposed in recent years to improve the detection^12,13^ and fragmentation of intact glycopeptides^14,15^. Unlike the regular proteome studies, computational tools for automated database searches and glycoprotein identification represent a major limitation^12^. Bioinformatics tools and search engines to automatically extract both glycan and peptide information remains the major hurdle in analyzing intact glycopeptide MS2 spectra. Some of the major problems with the proposed informatics tools include the lack of possibility to annotate both N- and O-linked glycopeptide spectra, high-throughput and batch mode analysis of large liquid chromatography-mass spectrometry (LC-MS) data sets^16–18^.

There is an urgent need of a user-friendly universal pipeline for profiling intact glycopeptides in a high-throughput and batch-wise manner. Here, we propose an approach using the widely used Mascot search engine for identification of intact N- and O-linked glycopeptides. This approach was first applied to standard glycoproteins, and further validated by a complex N-linked sialylated glycoproteome study of serum samples from control and prostate cancer patients.

## Results

### Defining glycan residues for Mascot database search

**Figure 1:**
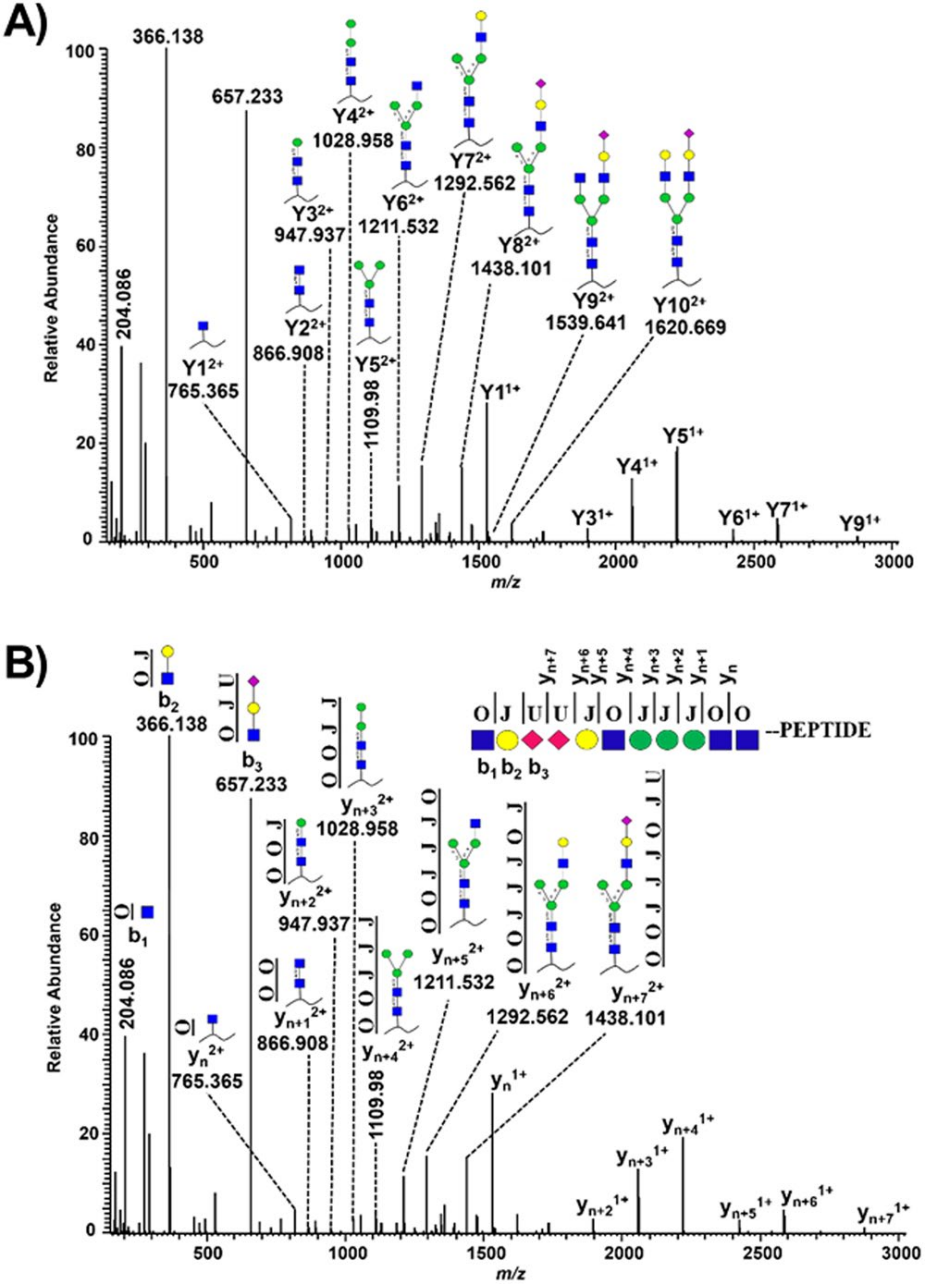
HCD-MS2 spectrum of a di-sialylated bi-antennary glycopeptide (*m/z* 1177.8137^3+^) derived from bovine alpha-1-acid glycoprotein 1. The glycopeptide was fragmented at an NCE value of 15. (**A**) Y_*n*_ represent the peptide bound glycosidic cleavage ions and the insert shows the corresponding peptide bound glycan structures. (**B**) Linearizing glycan structures with the corresponding three letters O (GlcNAc, GalNAc), J (Galactose, Mannose) and U (Neu5Ac) are depicted. A linearized di-sialylated bi-antennary glycan structure attached to the N-terminus of a peptide and annotated *y* and *b* type cleavage ions corresponds to the MS2 spectrum.

A series of Y type glycosidic fragment ions are required for confident characterization of glycan structures and both collision-induced dissociation (CID) and higher-energy collisional induced dissociation (HCD) fragmentation techniques provide such pattern at lower normalized collision energy (NCE) values. A typical example of an HCD (NCE = 15) MS2 spectrum of a glycopeptide derived from bovine alpha-1-acid glycoprotein is presented in Fig. 1A. Starting with the Y1 ion by following the mass differences between the most intense peaks and the mass difference between the Y10 ion and the precursor, the glycan sequence can be easily determined. The complete glycan structure contains four HexNAc_,_ five Hex and two Neu5Ac residues, which represent a di-sialylated biantennary N-glycan (Fig. 1A). Assuming the glycan residues similar to amino acids, deducing the glycan structure from glycopeptide MS2 spectrum is similar to peptide sequencing. In order to use the Mascot search engine for automated glycopeptide analysis, the basic requirements include that (i) the sugar residues must be defined with unique one-letter codes in a Mascot readable format, (ii) each glycan structure must be defined in a linear format and (iii) a customized database must be prepared which consists of combined protein and glycan sequences.

Mascot uses the Latin alphabet as one-letter codes and 20 of them are assigned to the standard amino acid residues, and B, X and Z are hard-coded. Of the remaining three letters (O, J and U), O was assigned to N-acetylhexosamine (GlcNAc, GalNAc), J to hexoses (Galactose, mannose) and U to sialic acid (Table 1). Fucose was defined as a variable modification on N-acetylhexoseamines (O). For Mascot, the three letters O, J and U must be defined in the unimod.xml file (Supplementary Fig. 1).

**Table 1:** Unique one letter codes in Mascot readable format, their definitions and residue masses of the four basic sugar types.

Glycan residues	Glycan type	Residue mass	One letter code
Gal, Man	Hexose	162.0528	J
GlcNAc, GalNAc	N-acetylhexoseamine	203.0794	O
Neu5Ac	Sialic acid	291.0954	U
Fuc	Deoxyhexose	146.0579	Variable modification on O

### Linearized glycopeptide sequences and custom glycoprotein databases

After manual annotation of various glycopeptide MS2 spectra, linear glycan sequences were defined based on the criteria that they should i) cover the maximum possible intense peaks in the MS2 spectrum and ii) provide close to complete information about the glycopeptide sequence. Considering the di-sialylated bi-antennary glycopeptide (Fig. 1A), the following linear sequence OJUUJOJJJOO-peptide fulfills the criteria mentioned above (Fig. 1B). By attaching the glycan sequence at the peptide N-terminus, the Yn type glycosidic cleavage ions now become the peptide cleavage type *y*_*n*_ ions. The last three residues at the N-terminus (OJU) cover the three most intense peaks of oxonium ions at 204.086 (HexNAc), 366.138 (HexNAc-Hex) and 657.233 (HexNAc-Hex-Neu5Ac) as *b*_1_, *b*_2_ and *b*_3_ ions. The remaining linear sequence (UJOJJJOO-peptide) can be annotated to the intense peaks as *y*_n_ to *y*_n+7_ ions (Fig. 1B). The spectrum now contains a series of eight *y* type and three *b* type intense ions. All major glycan structures were converted to linear sequences following the same principles (Supplementary Table 1). The next step was to create a customized database, where both the protein and glycan sequences co-exist. An in-house written python script was developed for this purpose (Supplementary File). Briefly, following an in-silico digestion, the tryptic peptides containing NxT/S/C motifs (N-linked glycosylation) or serine/threonine residues (O-linked glycosylation) and the linear glycan sequences were combined (Supplementary Fig. 2). The custom database used in the manuscript, if not otherwise described, consists of a total of 406 potential glycoproteins which were known to be glycosylated in serum (PeptideAtlas N-Glyco build 2010). After adding 21 unique linear sialylated glycan sequences (Supplementary Table 1), the database contained 41,727 potential glycopeptide sequences and a total of 1,195,485 residues.

### Identification of N- and O-linked glycopeptides by Mascot

**Figure 2:**
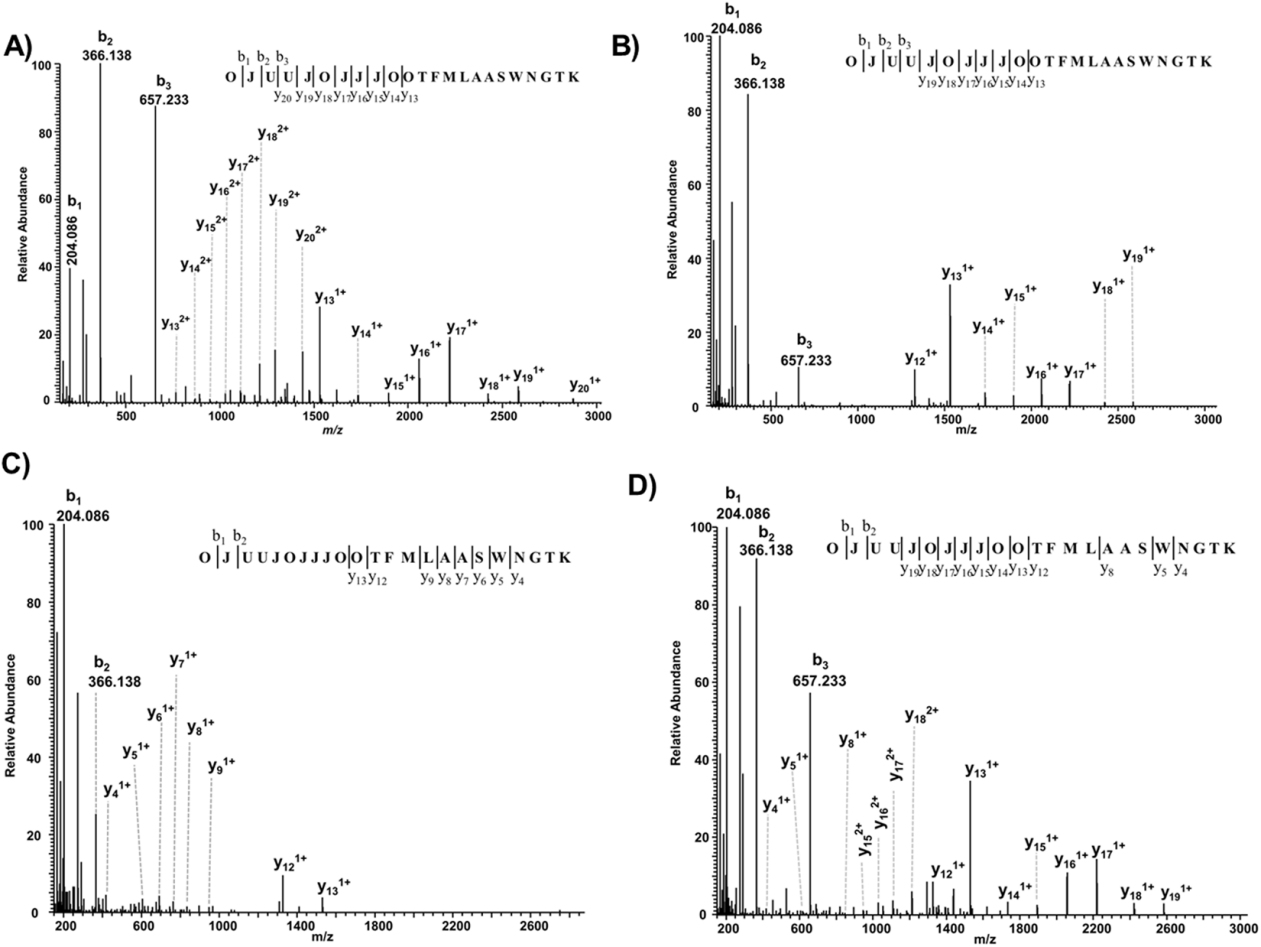
Mascot annotated MS2 spectra of a di-sialylated bi-antennary N-glycopeptide (*m/z* 1177.8137^3+^) fragmented at different NCE values. NCE values of (**A**) 15, (**B**) 25, (**C**) 35 and (**D**) the composite MS2 spectrum of the same precursor fragmented using the stepped NCE values of 15, 25 and 35 are displayed. Standard bovine alpha-1-acid glycoprotein 1 was digested with trypsin, the glycopeptides were analyzed by LC-MS and the data were searched against the custom glycoprotein database using the Mascot search engine.

The feasibility of the Mascot search engine for automated annotation of both N- and O-linked glycopeptides was validated using two standard bovine glycoproteins (alpha-1-acid glycoprotein and fetuin). When searched against the custom glycoprotein database, the MS2 spectrum shown in Fig. 1A is annotated as a di-sialylated bi-antennary N-glycopeptide of alpha-1-acid glycoprotein, with a Mascot ion score of 24 (Fig. 2A). As theoretically expected, Mascot annotated the intense peaks to a series of *y* ions starting from *y*_13_ (peptide + HexNAc) until *y*_20_ (peptide + HexNAc(O)_3_ − Hex(J)_4_ − Neu5Ac(U)_1_). Together with the precursor mass, and the *b*_1_*, b*_2_ and *b*_3_ ions, the presence of additional HexNAc(O)_1_ − Hex(J)_1_ − Neu5Ac(U)_1_ residues was confirmed, thereby providing 100% sequence coverage of the glycan (Fig. 2A). However, no other information in the spectrum confirmed the peptide sequence except the precursor mass. The lack of peptide fragmentation information in the MS2 spectrum might create difficulties in differentiating glycopeptide sequences resulting in similar Mascot ion scores. However, the fragmentation of the glycopeptides can be fine-tuned by the NCE values used for HCD fragmentation. As an example, the tryptic peptides of alpha-1-acid glycoprotein were fragmented at different NCE values. At NCE values of 15 and 25, the di-sialylated bi-antennary glycopeptide MS2 spectra displayed glycosidic fragment ions (Fig. 2A,B). However, the same glycopeptide contained a series of peptide cleavage type *y* ions (*y*_4_ to *y*_9_) at an NCE value of 35 with almost no information about the glycan structure. Hence, Mascot annotated mostly the peptide part of the glycopeptide sequence (Fig. 2C). Consequently, a single NCE value might not provide enough information about both the glycan and peptide sequence. With the stepped NCE option of quadrupole-orbitrap mass spectrometers, the instrument can acquire fragmentation data of the precursors at multiple collision energies. With this option, up to three different NCE values can be selected to generate a composite MS2 spectrum as shown in Fig. 2D, combining 15, 25 and 35 as NCE values. This MS2 spectrum revealed near to complete information about the glycan sequence and the peptide *y* ions (*y*_4_, *y*_5_, *y*_8_ and *y*_12_) were detected as well. Mascot unambiguously annotated this MS2 spectrum with an ion score of 34 (Fig. 2D).

**Figure 3:**
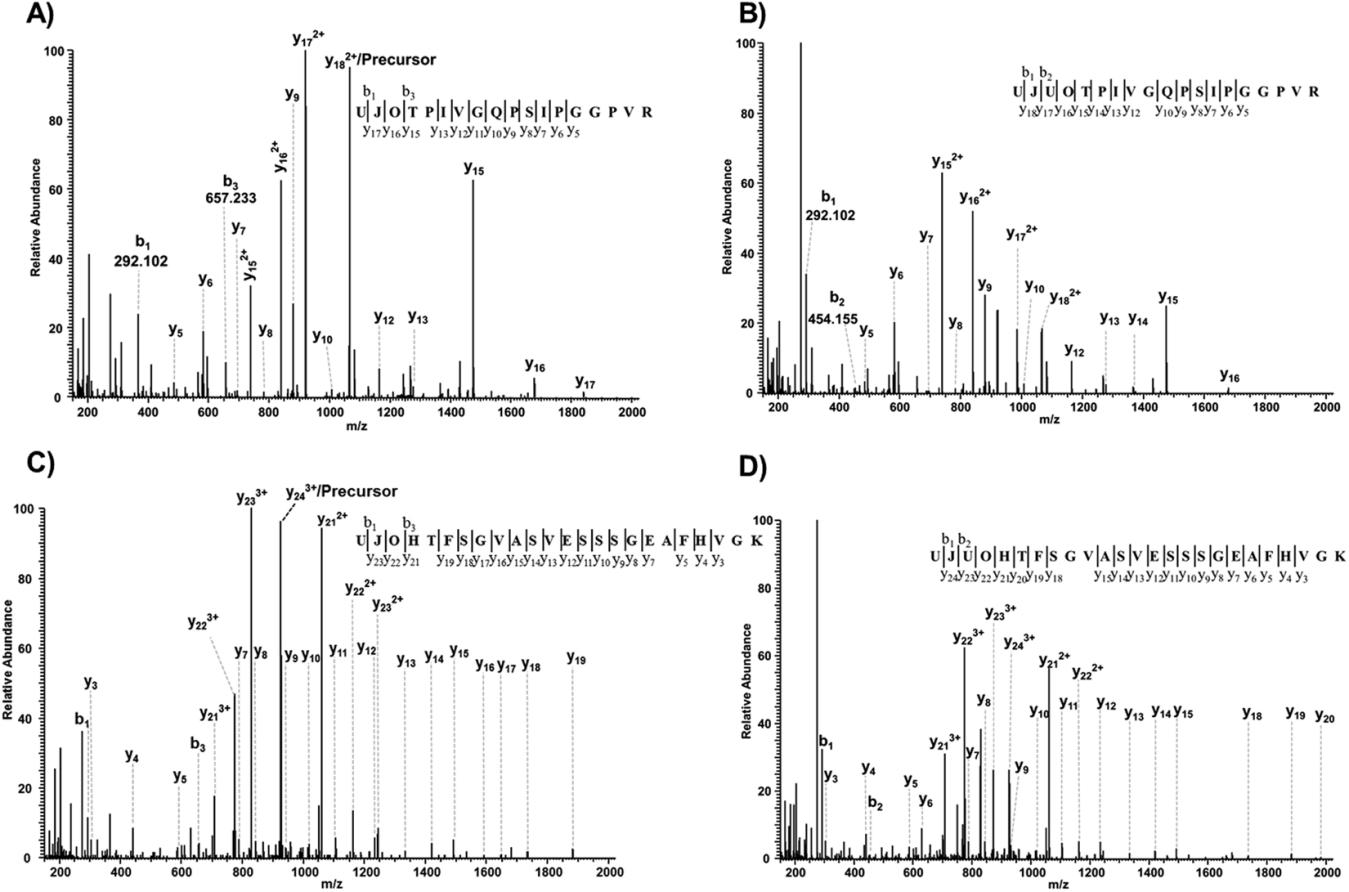
Mascot annotated O-glycopeptide MS2 spectra of fetuin using stepped NCE values. Bovine fetuin was digested with trypsin, analyzed by LC-MS using the stepped NCE function (15, 25 and 35) and searched against the custom O-glycoprotein database. Mascot annotated mono- (**A**,**C**) and di-sialylated (**B**,**D**) core-1 O-linked glycopeptide spectra from two different peptide sequences.

The feasibility of the Mascot search engine for the analysis of O-linked glycopeptides was validated by analyzing the mass spectrometry data of bovine fetuin against a custom O-glycoprotein sequence of fetuin. Mascot annotated mono- and di-sialylated core-1 O-glycans on two different peptide sequences. A series of *y* ions (*y*_5_ to *y*_18_) and *b* ions (*b*_1_, *b*_2,_ *b*_3_) covering the most intense peaks (Fig. 3A,B) clearly confirmed that these MS2 spectra correspond to the O-linked glycopeptides. These spectra were unambiguously annotated with an excellent Mascot ion score of more than 40. Similar to N-linked glycopeptides, the *b*_1_, *b*_2_ and *b*_3_ ions at *m/z* values of 292.102 (Neu5Ac), 454.155 (Neu5Ac-Hex) and 657.233 (Neu5Ac-Hex-HexNAc) covered the low mass glycan fragment ions and provided an additional layer of confirmation about the O-glycopeptide spectra. A similar fragmentation behavior was observed for two other O-glycopeptides of the same protein (Fig. 3C,D). To further display the feasibility of Mascot, analysis of bacterial O-glycosylation was performed on a purified PilE protein. The PilE protein contains a di-N-acetyl-bacillosamine (diNAcBac) and galactose based glycans with a potential acetylation on the galactose residue^19^. Mascot was able to annotate the diNAcBac (Supplementary Fig. 3A), diNAcBac-Gal (Supplementary Fig. 3B) residues as well as the monoacetylation (Supplementary Fig. 3C) and diacetylation (Supplementary Fig. 3D) on galactose residues. For the complex O-glycosylation study, we re-analyzed the previously published mass spectrometry data^20^ from the immunoaffinity purified fractions and whole cell extract of a *Neisseria gonorrhoeae* strain. The Mascot annotated glycopeptides were compared to the previously published data, where software assistance and manual data analysis was performed^20^ and the majority (11/13 glycopeptides) of the previously confirmed O-glycopeptides were identified automatically (Supplementary Table 2). Taken together all these results clearly indicates the potential of the described approach i.e. using stepped NCE values, a custom linearized glycoprotein database and the Mascot search engine for automated glycopeptide annotation.

### Identification and label-free quantification of serum N-glycoproteome

**Figure 4:**
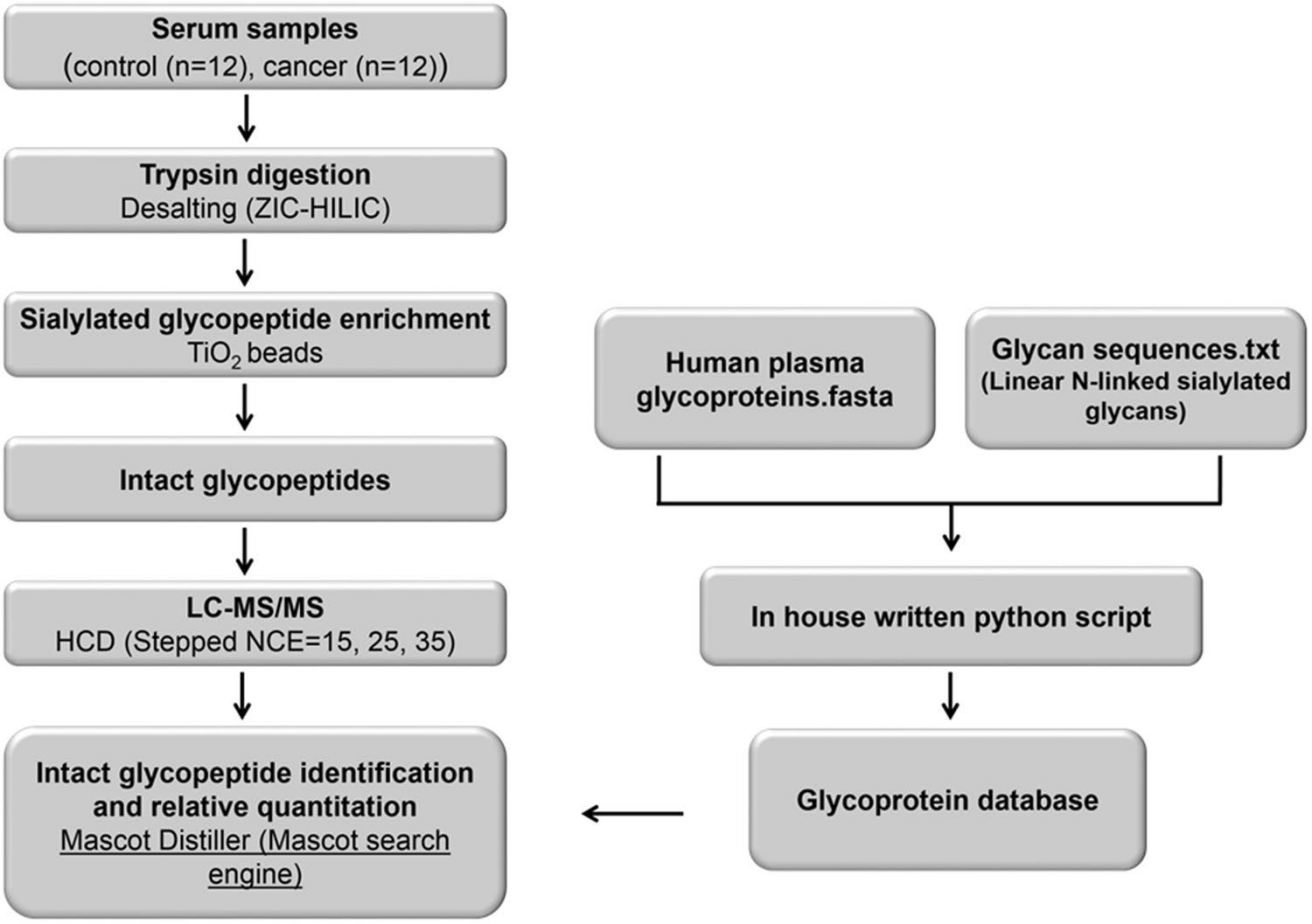
Workflow for N-glycoproteome analyses of serum samples. Briefly, the serum samples from control and patients diagnosed with prostate cancer were digested with trypsin and desalted with ZIC-HILIC SPE. Next, the N-linked sialylated glycopeptides were enriched with TiO_2_ beads, followed by LC-MS analysis using a Q Exactive mass spectrometer applying stepped NCE for HCD fragmentation. The intact glycopeptide mass spectra were submitted to the Mascot search engine for identification and relative quantification with Mascot Distiller. The data was searched against a custom glycoprotein database prepared from 21 linear N-linked sialylated glycans and proteins (444) known to be glycosylated in serum (PeptideAtlas N-Glyco build 2010).

The proposed procedure was validated by analyzing the N-linked sialylated glycoproteome of serum samples from healthy individuals (n = 12) and patients diagnosed with prostate cancer (n = 12). Tryptic peptides from serum samples were desalted using zwitterionic chromatography-hydrophilic interaction liquid chromatography solid phase extraction (ZIC-HILIC SPE) to enrich glycopeptides, followed by enrichment of sialylated glycopeptides with TiO_2_ beads^21,22^ (Fig. 4). The N-linked sialylated glycopeptides were analyzed by LC-MS using HCD with stepped NCE and the acquired MS2 spectra were submitted to the Mascot search engine for automated identification and relative quantification using Mascot Distiller (Fig. 4).

**Figure 5:**
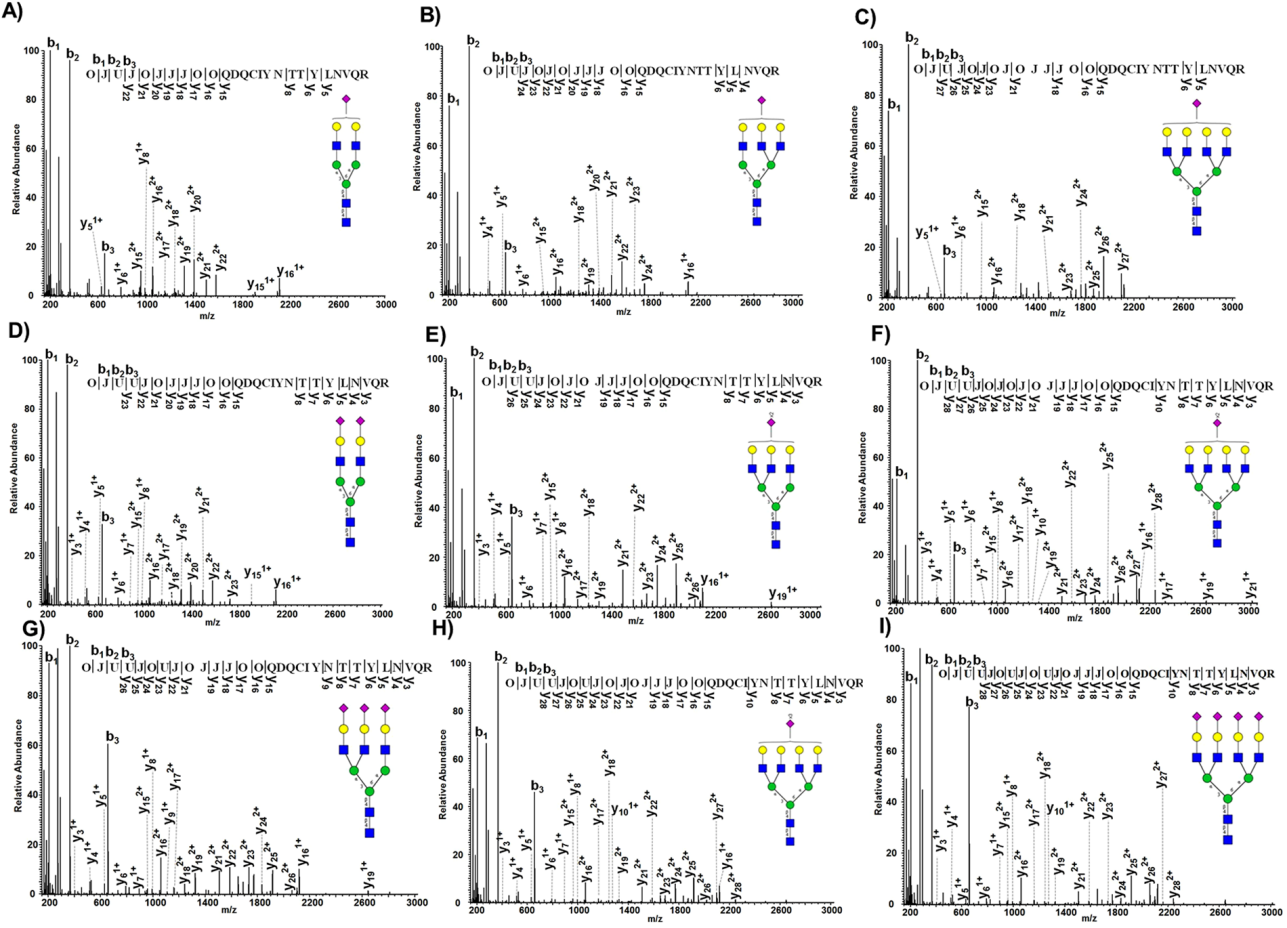
Annotation of nine different glycan structures with varied degree of complexity and sialylation by Mascot on a single glycosylation site (Asn 93) of alpha-1-acid glycoprotein 1 in serum. Shown here are the representative HCD MS2 spectra annotated by Mascot. The nine different glycopeptide variants included the mono-sialylated bi- (**A**), tri- (**B**), tetra-antennary (**C**), and the di-sialylated bi- (**D)**, tri- (**E**), tetra-antennary (**F)**. Tri- (**G**,**H**) and tetra-sialylated (**I)** glycan structures on the same glycosylation site were also annotated by Mascot.

The result of the described strategy for large scale automated glycopeptide analysis of LC-MS datasets was demonstrated by serum alpha-1-acid glycoprotein 1 (A1AG1) as an example. Considering zero missed cleavages, NxT/S/C motifs and a peptide length of 6–30 amino acids, A1AG1 potentially contained two N-glycosylation sites in the custom glycoprotein database (QDQCIYNTTYLNVQR, ENGTISR). A1AG1 was identified with a protein score of 1559 and 27% sequence coverage by the database search of 24 LC-MS runs. Most of the sialylated N-glycans were identified on the sequence QDQCIYNTTYLNVQR. Mascot annotated nine different mono-, di-, tri- and tetra-sialylated N-glycan structures on this glycosylation site (Fig. 5). Almost all the intense peaks in the MS2 spectra of mono-sialylated bi- (Fig. 5A), tri- (Fig. 5B) and tetra-antennary (Fig. 5C) glycopeptides were annotated by Mascot, confirming the presence of these glycan structures. The peptide sequence was confirmed by annotation of y5, y6 and y8 ions. MS2 spectra shown in Fig. 5 D–F were annotated to di-sialylated bi-, tri- and tetra-antennary glycopeptides, confirmed by a complete series of y ions representing both peptide and glycan cleavages. The same was found for tri- and tetra-sialylated glycan structures on the same peptide sequence (Fig. 5G–I). Nine different glycan structures with varied degree of complexity and sialylation on the same glycosylation site, and near to complete information about both the peptide and glycan part proved the capability of the current approach for large scale automated glycopeptide analysis. Some of the above sialylated glycopeptides were also identified with attached fucose residues. As mentioned above, fucose was considered as a variable modification during the database search. Though it is not possible to pinpoint the exact location of fucose residues, it can be easily concluded whether the fucose is attached to the core HexNAc residue or the HexNAc residues after the trimannosyl core glycan structure. For example, the top scoring matches of tri-sialylated tri-antennary and di-sialylated tetra-antennary glycopeptides indicated a fucose residue after the core structure. The absence of peak at +146 Da following the peptide + HexNAc peak clearly indicated that the fucose residue is not attached to the core HexNAc (Supplementary Fig. 4A,B). As opposed to the above examples, Mascot annotated the fucose residue to the core HexNAc of a di-sialylated bi-antennary glycopeptide of alpha-2-macroglobulin. The presence of a peak at +146 Da, following the peptide + HexNAc peak clearly indicated that the fucose is attached to the core structure (Supplementary Fig. 4C). Therefore, it must be considered that the fucose is either attached to the core HexNAc or HexNAc residues following the core glycan structure when determining the position of fucose residues in Mascot output. In addition to fucose, other modifications such as sulfation and phosphorylation of HexNAc or Hex could also be considered as variable modifications if this is of interest. However, using more variable modifications increases the search space and thus the uncertainty in some assignments.

**Figure 6:**
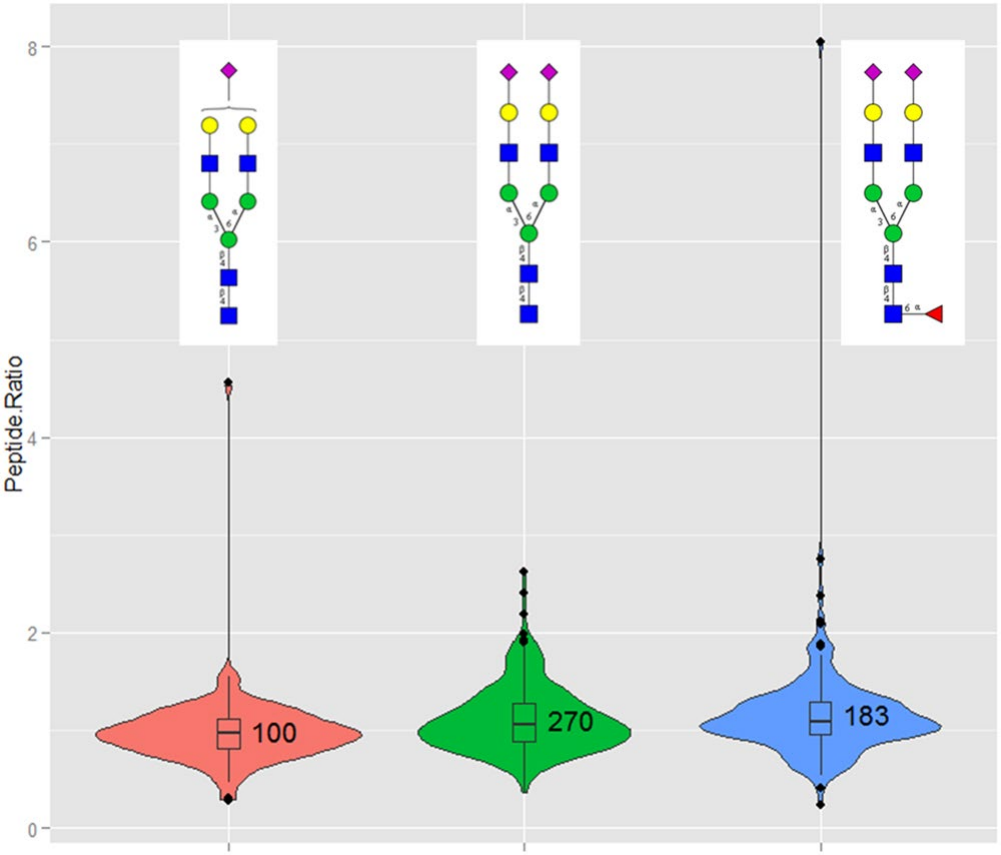
Violin plots representing the glycopeptide ratios (aggressive vs. indolent prostate cancer) of the three most frequent glycopeptide variants identified in the current study. Glycopeptides identified and quantified in 24 serum samples were segmented based on the glycan structures irrespective of the protein origin. The three most frequent glycopeptide variants were the mono-sialylated bi-antennary, di-sialylated bi-antennary without and with one fucose residue. Mascot Distiller was used to calculate the XIC values and the corresponding ratios between aggressive (12) and indolent (12) samples. Glycopeptide precursors contributing to a minimum 50% of the XIC peak area and passing the correlation threshold of 0.8 were only considered.

Using this approach, a total of 257 glycoproteins were identified from the 24 serum samples (Supplementary Table 3). Within these 257 glycoproteins, a total of 970 unique glycosylation sites and 3447 non-redundant glycopeptide variants were identified (Supplementary Tables 4, and 5). Of these 3447 glycopeptide variants, the most abundant are the di-sialylated bi-antennary glycans with no (377), one (291) and two fucose residues (169). The next major glycopeptide variants included the di-sialylated tri-antennary and mono-sialylated di-antennary glycopeptide variant without and with fucose residues (Supplementary Table 5). The specific enrichment for di-sialylated bi-antennary glycans might indicate the abundance of these glycans in the serum proteins. However, an effect of the enrichment protocol cannot be ruled out. Label-free quantification of the glycopeptides (aggressive vs. indolent prostate cancer) was performed using the replicate quantitation protocol of Mascot Distiller. The median protein ratios revealed no significant changes between aggressive and indolent samples and most of the protein ratios were within the range of 1.0 ± 0.5 (Supplementary Table 3). To find out any quantitative differences at the glycosylation level, the glycopeptides were segmented based on the glycan structures irrespective of the protein origin and the corresponding ratios were plotted as violin plots. Figure 6 displays the glycopeptide ratios of the three most abundant glycan structures and most of them have peptide ratios near to 1.0, indicating no significant changes between the analyzed indolent and aggressive cancer samples. Glycopeptide ratios of various other glycan structures which were identified in more than 10 different peptide sequences are presented in Supplementary Fig. 5. Most of these structures had also peptide ratios around 1.0 with a very few being up or down. For example, the median glycopeptide ratio of the tri-sialylated tri-antennary glycopeptides is near 1.0 based on 77 values, whereas the mono- (73 values) and di-fucosylated (19 values) versions have a median peptide ratio slightly above 1.0. The tri-sialylated tetra-antennary glycopeptides had two different populations at median peptide ratios of 1.0 and 1.5, whereas the fucosylated version had a median peptide ratios slightly above 1.0 (Supplementary Fig. 5). Summarized, the presented data shows the ease and feasibility of the proposed workflow for automated glycopeptide identification and quantification.

In addition to the database used in obtaining the above presented results, the LC-MS data sets of the 24 serum samples were also searched against differentially sized custom glycoprotein databases created from (i) all known plasma/serum proteins from PeptideAtlas build 2010 (2421 glycoproteins), (ii) all deamidated proteins identified following PNGaseF treatment of glycopeptides from the same 24 serum samples (280 glycoproteins) and (iii) Swiss-Prot annotated human proteome (14120 glycoproteins). Irrespective of the databases, 68 glycoproteins were consistently identified in all four different databases (Supplementary Fig. 6). There is a good level of agreement between the three plasma protein related databases because 121 glycoproteins were consistently identified. The deamidated proteins (280 proteins) identified after the PNGaseF treatment potentially represent well the detectable glycoproteins present in the 24 serum samples. Comparing the glycoprotein databases created from deamidated proteins identified following PNGaseF treatment and plasma glycoproteins reported in Peptide Atlas, out of the 257 glycoproteins identified, 163 were found to be common representing 63% overlap (Supplementary Fig. 6). This result clearly indicates the authenticity of the glycoproteins identified by the workflow presented in this study.

## Discussion

A large variety of informatics tools have been developed for automated glycopeptide analysis which advanced the glycoproteomics field^23^. However, recent reviews^16,17^ summarizing the glycoproteomics field in terms of available software tools suggested the need of a single software tool which could address the following concerns: (i) elucidation of both N- and O-linked glycopeptide spectra, (ii) matching glycopeptides to known protein sequences, (iii) scoring/ranking of potential glycopeptides, (iv) usage of product ion spectra, and (v) high-throughput and batch-wise analysis. In this report, we addressed these concerns by using the widely applied Mascot search engine for automated glycopeptide analysis. In principle, other protein search engines could be used as well, if additional letters can be defined for monosaccharides as described here.

The success of the software-assisted intact glycopeptide analysis also depends on the enrichment strategy^21^ and the information available in the MS2 spectra. The enrichment strategy employed in this study worked well to enrich sialylated glycopeptides and the LC-MS data sets contained mainly glycopeptide spectra. For any software tool, the MS2 spectra of intact glycopeptides should contain both peptide and glycan information in order to provide a scoring and ranking of potential glycopeptide identifications and matching the glycopeptides to protein sequences. A considerable amount of research has been performed for developing efficient fragmentation tools for glycopeptide analysis. Unlike the collision based fragmentation techniques, the glycan structure remains relatively intact in electron transfer dissociation (ETD) spectra^24^, thus providing information about the peptide sequence. The combination of collision based (HCD/CID) and electron transfer (ETD/ECD) based fragmentation techniques provide complementary information about the glycopeptide sequences^25^. Data driven acquisition strategies, for example HCD-product dependent CID-and ETD fragmentation strategies have also been shown to be effective in intact glycopeptide analysis^26–29^. The recently introduced electron transfer and higher-energy collision induced dissociation (EThcD) technique seems to work quite well for intact glycopeptide analysis^30–32^. However, with the used Q Exactive mass spectrometer, we could only use HCD. Therefore, we showed the advantages of using stepped HCD^33^ while generating glycopeptide MS2 spectra. HCD mass spectra at lower energies (Fig. 2A,B) are typically dominated by glycosidic fragment ions, whereas at higher energies the mass spectra (Fig. 2C) mainly contained peptide cleavage ions, thereby hampering successful mapping of both glycan and peptide moieties. The HCD mass spectra using stepped NCE provided information both at the glycan and peptide level (Fig. 2D). A recent study also showed the same effect using low and high energy CID on a Q-TOF instrument for synthetic glycopeptides and standard glycoproteins^18^.

A large number of available software tools for glycopeptide annotation deals mainly with N-linked glycosylation. Software tools that can automatically annotate both N-linked and O-linked glycopeptides are of great advantage. For example, Mascot annotated a total of nine different mono-, di-, tri- and tetra-sialylated N-glycan structures on a single glycosylation site (Asn 93) of serum alpha-1-acid glycoprotein 1 (Fig. 5). Though, the sialylated N-linked glycans were the main focus in this study, the presented Mascot approach can of course identify other types of N-glycan structures **(**Supplementary Fig. 7**)**. O-linked glycosylation on the other hand is more difficult to study, due the inherent lack of a consensus motif. The obtained results using bovine fetuin documented that the Mascot search engine can indeed be used for O-linked glycopeptide analysis. Mono- and di-sialylated core-1 O-linked glycans were annotated to two different sequences. According to UniProt and some recent publications^34^, the peptide sequence HTFSGVASVESSSGEAFHVGK carries only phosphorylation on serine residues (320, 323 and 325). However, the data presented here (Fig. 3C,D), clearly indicated to the presence of mono- and di-sialylated O-linked glycans on this peptide sequence. Due to the lack of a consensus glycosylation motif, while assembling the O-glycopeptide database, every serine and threonine peptide must be considered as a potential glycopeptide, thus challenging the large-scale O-glycoproteomics studies.

The established approach was further validated by analyzing LC-MS data sets generated from 24 serum samples. Mascot annotated a total of 257 glycoproteins containing 4653 redundant N-linked sialylated glycopeptide variants with an estimated false discovery rate (FDR) of 8%. The FDR estimation for intact glycopeptide identifications is debatable and especially in case of glycopeptide identifications in relatively small numbers, the accurate estimation of FDR values is not possible. Moreover, FDR control of both glycan and peptide identifications is challenging and based on the analytical workflow used, some customized strategies have been proposed^35,36^. Provided fragmentation information of both peptide and glycans of all the glycopeptides, FDR tools provided in Mascot can be confidently used. Therefore, we only considered positive hits if a Mascot ion score of 25, a top scoring match to a particular spectrum and a significance threshold *p-value* < 0.001 was achieved. At this point, we suggest using more confident filters such as the significance threshold *p-*values/Mascot ion scores. Moreover, it was even possible to extract the XIC values and quantitatively compare the glycopeptide identifications using Mascot Distiller. The protein as well as the glycopeptide ratios indicated very little to no significant differences between indolent and aggressive serum prostate cancer samples. Still, we showed here the possibility of high-throughput identification and relative quantification of intact glycopeptides using this large dataset of 24 LC-MS runs. Due to the availability of well-established tools like Mascot Daemon, Mascot Distiller and Proteome Discoverer, relatively fast identification and comparison of multiple LC-MS glycopeptide data sets is possible. Many of the available software tools for glycoproteomics lack this ability of high-throughput and batch-wise analysis of large datasets.

Despite the significant results obtained with this approach, some issues regarding intact glycopeptide analysis are yet to be solved and are worth discussing. The majority of the glycopeptide identification strategies consider the glycan structures as monosaccharide compositions, whereas we defined in our approach the glycan structures as linear sequences that best represents their behavior in the glycopeptide MS2 spectra. With any of these approaches, it is difficult to analyze glycan structures for example specifying linkage information and differentiating glycan topologies. Manual interpretation of the MS2 spectra, in particular spectra of the glycans alone probably is the best way in such special cases. With our approach, for example the presence or absence of fucose residues can be specified without prior knowledge. Moreover, as shown in the results (Supplementary Fig. 4), no prior knowledge is required in defining the position of fucose residues, as Mascot automatically annotates the fucose residue to the core HexNAc or HexNAc residues following the core glycan structure. In terms of differentiating glycan topologies, if these topologies exhibit different fragmentation behavior, this could be specified in the linear glycan sequences and thereby enabling the possibility of topology differentiation. However, this should be experimentally verified and manual validation will still be required for confirmation. The N- and O-glycan databases used in this study are relatively small. Since, the samples were specifically enriched for sialylated glycans, the N-glycan databases used in the study considered only sialylated glycans. Using the total human proteome and glycome databases in preparing custom glycoprotein databases would of course have an impact on the quality of assignments. For example, keeping a constant glycan database and using varying sizes of the proteome databases, the obtained results (Supplementary Fig. 6**)** indicated that the overlap was much higher between focused plasma protein databases, compared to the whole human proteome. A recent study scrutinizing the frequently used glycopeptide identification Byonic software, also indicated that the glycome size, proteome size and number of modifications can have a profound impact on the search outcome^37^. This indeed is a well-known observation, even with the regular proteome search engines, that the database size and number of variable modifications increases the search space exponentially thus influencing the search outcome. Considering the complexity involved in glycoproteomics, at this point we suggest using the custom glycoprotein databases that closely represent the samples used in the study. Iterative search approaches, for example provide an alternative opportunity to overcome this limitation. The data could be searched against the database containing only N-linked sialylated glycans for the first search. The unannotated MS spectra can then be searched against another database of other glycans of potential interest and this could be iteratively repeated. Though the stepped HCD function used in the study provided both peptide and glycan information, we observed that this is not universal and for some glycopeptide sequences, no peptide fragmentation was observed. We believe that this also has an impact on the search outcome, when utilizing larger glycoproteome databases. Fragmentation methods that generate glycan and peptide fragments, irrespective of glycopeptide sequences, will open up the possibility of using general proteome databases. One specific limitation applicable for the described approach is that since the glycan compositions are added to the peptide N-terminus, the peptide b-ions present in the spectrum cannot be used. The confirmation of the peptide sequence arises only from the y-ions, which are typically dominant in tryptic peptides using CID and HCD.

As mentioned above, several computational tools have been developed for automated identification of glycopeptides and the following reviews provide a detailed overview^16,17,25,38,39^. A large number of academically developed computational tools showed potential on automated glycopeptide identification studies for example, GlyDB^40^, GlyPID^41^, GlycoFragWork^42^, GlycoMaster DB^43^, GlycoPeptideSearch^44^, GlycoPep Detector^45^, GlycoPep Evaluator^46^, GlycoPep Grader^47^, Integrated Glyco- Proteome Analyzer^48^, MAGIC^49^, pGlyco^35^, Protein Prospector^50,51^, SweetNET^52^, Sweet-Heart^53^ and a few more^17,25^. Most of the academic tools are usually open-source, however academic tools are mostly designed for specific needs and the majority of the tools are not continually followed up. Moreover, very often they are lacking an appropriate graphical interface making them less user-friendly and often need additional informatics assistance to utilize them. Commercial tools on the other hand are designed to be user friendly, continuously developed further and upgraded based on the research demands. SimGlycan^54^, GlycoQuest and Byonic^55^ are among the commercially available glycopeptide identification tools.

SweetNET^52^, a recently introduced bioinformatics workflow uses an iterative process where glycan derived oxonium ion are used to filter the MS2 data for glycopeptides, the resulting set is then searched against protein databases to generate molecular networks for intact large scale glycopeptide identification. For the database search of N-glycopeptides using Mascot, the glycan variable modification was defined as 5Hex + 4HexNAc attached for asparagine residues and loss of 5Hex + 4HexNAc or 5Hex + 3HexNAc from b- and y-ions including the N-glycosylation site was included. Despite using the same search engine, the general concept of SweetNet is completely different to our approach. Byonic is one of the most frequently used software package for glycopeptide data analysis and successfully reported in several different glycoproteomics studies^56–59^. Byonic identifies glycopeptides at the level of peptide sequence and glycan composition by searching the predefined or user-defined separate glycan and protein databases. Glycan residues are specified as monosaccharide compositions and the potential glycopeptide candidates are scored by placing each glycan on the consensus N-glycosylation motifs. In addition to the peptide/glycopeptide fragments, the presence of common oxonium ions and glycopeptide ions (Pep + HexNAc) are also considered while scoring the glycopeptides. In our approach unlike Byonic, first glycan structures are defined in a linear fashion, which at best represent their behavior in the MS2 spectra. The linear glycans and the protein sequences are then curated into a single glycoprotein database. Fragmentated glycopeptides are searched against this database and scored based on the peptide type *b-* and y-ions using the standard Mascot scoring algorithm. When comparing the Byonic software with our approach, quite similar results were obtained. Some of the recent large-scale glycoproteomics studies also displayed the successful identification of thousands of glycopeptides^56,58,60^. However, one of the major advantages of using Mascot for automated glycopeptide analysis is its wide distribution and easy to use nature compared to many of the available software tools for glycoproteomics analysis. Mascot as a computational tool is continuously followed up since two decades, widely acclaimed and established in the proteomics community across the world and easily adaptable for glycopeptide analysis as described here. The necessary changes to establish Mascot for glycopeptide analysis are simply done by defining the letters (O, J, U) in the unimod.xml file (Supplementary Fig. 1) and updating the Mascot server with the glycoprotein database. The linear glycan sequences as well as the script to prepare a custom glycoprotein database are presented along with this report, and just needs to run a single command before the database is ready. Thus, no specific informatics skills are required to establish this workflow and a typical single LC-MS file from e.g. serum need a couple of minutes until the glycopeptide identifications are obtained.

In conclusion, we showed that Mascot, a widely accepted and used software could be easily implemented for automated glycopeptide analysis. Though, at this point it does not solve all the problems associated with glycoproteomics, this single tool collectively allows the (i) elucidation of both N- and O-linked glycopeptide spectra, (ii) matching glycopeptides to known protein sequences, (iii) scoring and ranking of potential glycopeptides, (iv) usage of product ion spectra, and (v) high-throughput and batch-wise analysis.

## Methods

### Materials

Acetonitrile (MS grade) was purchased from Burdick Jackson (Seelze, Germany). Acetone, ammonium bicarbonate, ammonium hydroxide, dithiothreitol (DTT), formic acid, iodoacetamide (IAA) potassium hydrogen phosphate and trifluoroacetic acid (TFA) were bought from Sigma-Aldrich (Oslo, Norway). Sequencing grade modified trypsin was obtained from Promega (Madison, WI, USA). Phosphate buffered saline (PBS) was obtained from Life Technologies (Oslo, Norway). TiO_2_ beads were obtained from GL Sciences Inc (Japan). Glycolic acid and pyrrolidine were purchased from Merck KGaA (Darmstadt, Germany).

Serum samples from 24 participants were kindly provided by the Janus Serum Bank (owned by the Cancer Registry of Norway)^61,62^. Blood samples were obtained from 24 non-fasting participants, serum was separated following standard methods, and all samples were stored at −25 °C. Within the 24 blood samples,12 samples were collected from men diagnosed with localized prostate cancer and no prostate cancer specific fatality during the follow-up (median 8,1 year), and were age-matched with 12 serum drawn from patients diagnosed with high risk/metastatic prostate cancer that would later encounter prostate cancer specific mortality within 3 years (median 2,2 years). The Janus Serum Bank has not informed written consent from the donors before 1990 because it was not required at that time. However, we have the license to use the samples in research from The Norwegian Data Protection Authority, and the study was approved by the Regional Committee for Medical and Health Research Ethics (REC) (2010/593 REC South-East).

The *Neisseria gonorrhoeae* PilE protein and the whole cell extract were obtained from previously published studies^19,20,63^. Briefly, *N. gonorrhoeae* cells were harvested from 20 plates incubated for 18–20 h at 37 °C, resuspended in 10 mL of buffer A (PBS pH 7.8, 0.1% Triton X-100) with protease inhibitors (1 × Complete protease inhibitor mixture EDTA-free (Roche)), and lysed by sonication. The cell suspensions were centrifuged at 20,000 g for 20 min at 4 °C to remove debris and unbroken cells, and the supernatants were kept as whole cell lysates. For immunoprecipitation, 100 µL of npg1 (1 mg/mL) (mAb recognizing a diNAcBac monosaccharide-associated epitope) was incubated with protein G-sepharose (2 mg/mL, Sigma Aldrich) overnight at 4 °C with rotation. The beads were washed three times with buffer A, 5 ml of whole cell lysate was added to the protein G-sepharose bound mAb, and the sample was incubated at 4 °C with rotation overnight. After washing three times with buffer A containing 1 M NaCl, the immunopreciptated proteins were eluted with 50 μL of 100 mM glycine buffer (pH 2.4) incubated with rotation for 30 min at 4 °C. The glycine buffer elution step was repeated three times.

### In-solution tryptic digestion

The protein content in serum samples (20 µL), was precipitated overnight with acetone (−20 °C, 200 μL). The samples were then centrifuged (13,000 g) and the supernatant was discarded. Protein pellets were then resuspended in 200 µL with 50 mM ammonium bicarbonate buffer, to which 10 μL of 200 mM DTT was added to reduce the disulfide bridges (30 min, 60 °C). Thiols were then alkylated with 30 µL of 100 mM IAA (1 h, 37 °C, dark), following with 30 µL of 200 mM DTT (30 min, 37 °C) to quench IAA. The protein content in the samples was further diluted with 50 mM ammonium bicarbonate buffer (200 μL) and digested with trypsin (10 µg) for 16 hours at 37 °C. The digestion was terminated by adding 5 µL of 50% formic acid. The samples were then vacuum concentrated (Eppendorf, Germany), dissolved in loading/washing buffer (80% acetonitrile, 1% TFA) and desalted using ZIC-HILIC cartridges (SeQuant®). The peptides were eluted from the cartridges with aqueous 1% TFA and vacuum concentrated. The purified PilE protein and the whole cell extract from *Neisseria gonorrhoeae* strain was also prepared using the same procedure as above.

### Enrichment of sialylated glycopeptides from serum

Sialylated glycopeptides from serum were enriched with TiO_2_ beads^21^, which were washed initially in loading buffer (70% acetonitrile, 1% TFA, 1 M glycolic acid) for three times. The dried tryptic peptides were resuspended in 500 μL of loading buffer (70% acetonitrile, 1% TFA, 1 M glycolic acid), to which 2 mg of TiO_2_ beads as a slurry was added and incubated for one hour under continuous shaking. The supernatant was collected, 2 mg of fresh TiO_2_ beads were added and incubated for another hour. Beads from two incubations were pooled together and gently washed with 200 μL of loading buffer. The TiO_2_ beads in loading buffer (100 μL) were then transferred into 200 μL pipet tips packed with C18 disks and washed one more time with 100 μL of loading buffer. Bound peptides were sequentially eluted with 50 μL each of 0.5 M K_2_HPO_4_, 10% ammonium hydroxide, 5% pyrrolidine and finally with 100 μL of 10% ammonium hydroxide in 50% acetonitrile. Eluted peptides were acidified by adding 2 μL 50% formic acid and the sample was vacuum dried and cleaned with ZipTip-C18 (Millipore, Billerica, MA, USA).

### Mass spectrometry

The dried peptides were dissolved in 25 µL of aqueous 2% acetonitrile containing 1% formic acid and 5 µL of sample was injected into a Dionex Ultimate 3000 nano-UHPLC system (Sunnyvale, CA, USA) coupled online to a Q Exactive mass spectrometer (ThermoScientific, Bremen, Germany) equipped with a nano-electrospray ion source. For liquid chromatography separation, an Acclaim PepMap 100 column (C18, 3 µm beads, 100 Å, 75 μm inner diameter, 50 cm) was used. A flow rate of 300 nL/min was employed with a solvent gradient of 3–10% B in 2 min, to 50% B in 110 min and then to 80% B in 2 min. Solvent A was 0.1% formic acid and solvent B was 0.1% formic acid in 90% acetonitrile. The mass spectrometer was operated in the data-dependent mode to automatically switch between MS and MS/MS acquisition. Survey full scan MS spectra (from m/z 400 to 2000) were acquired with the resolution R = 70,000 at m/z 200, after accumulation to a target of 1e6. The maximum allowed ion accumulation times were 100 ms. The sequential isolation of up to the seven most intense ions, depending on signal intensity (intensity threshold 5.6e3) were considered for fragmentation using higher-energy collisional induced dissociation (HCD) at a target value of 100,000 charges and a resolution R = 17,500 with stepped NCE 15, 25 and 35. Target ions already selected for MS/MS were dynamically excluded for 30 sec. The isolation window was m/z = 2 without offset. The maximum allowed ion accumulation for the MS/MS spectrum was 180 ms. For accurate mass measurements, the lock mass option was enabled in MS mode for internal recalibration during the analysis.

## Data analysis

### Custom glycoprotein database

To create a custom glycoprotein/peptide database we have used an in-house written python based script (Supplementary File). The script requires protein sequences in FASTA format and linearized glycan sequences in a text file format as input and it automatically generates a custom glycoprotein/peptide FASTA file, which can be then uploaded to Mascot. Briefly, an in silico digestion is performed considering trypsin as endoproteinase and zero missed cleavages. Peptides with a minimum length of five amino acids and maximum length of thirty amino acids were considered further. All the tryptic peptides were then scanned for NxT/S/C motifs (N-linked glycosylation) or serine/threonine residues (O-linked glycosylation) and only these peptides were considered further (Supplementary Fig. 2). The linearized glycan sequences from the text file will then be added to all the NxT/S/C or the serine/threonine containing peptides. The peptide and glycan were attached at first core HexNAc residue and the peptide N-terminus. For example by assuming four glycan sequences, then the NxT/S/C peptide appears with four different glycopeptide sequences. The script also generates a peptide centric database where each of the glycopeptide is considered as a single entry (Supplementary Fig. 2). Users have the option to use either the protein or peptide centric database. For standard glycoprotein analysis, the proteins of interest (bovine alpha-1-acid glycoprotein 1, fetuin) and either the 21 unique linear sialylated glycans (N-glycosylation) or the two different mono- and di-sialylated core-1 O-glycans (O-glycosylation) were used for the custom databases. The PilE protein data was searched against a glycoprotein database generated using the *Neisseria gonorrhoeae* MS11 protein database and diNAcBac, galactose based glycans (4427 sequences, 1,500,668 residues). For large scale O-glycosylation study of *Neisseria gonorrhoeae strain*, the *Neisseria gonorrhoeae* MS11 protein database, mono- and di-diNAcBac based glycans were considered for the custom glycoprotein database (4442 sequences, 1,825,781 residues). For large-scale serum glycosylation analysis, the custom glycoprotein database was prepared using a total of 444 glycosylated serum proteins (PeptideAtlas N-Glyco build 2010) and a list of 21 unique linear sialylated glycan sequences were attached to all the NxT/S/C peptides. Of these 444 glycoproteins, a total of 406 proteins contained NxT/S/C peptides with zero missed cleavages and peptide length between five and thirty amino acids.

### Identification of N- and O-linked glycopeptides/proteins

The raw LC-MS data sets from standard glycoproteins were processed to generate peak list in Mascot generic format (*.mgf) using ProteoWizard release version 3.0.331. The peak lists were then searched against the custom N- or O-linked glycoprotein databases using Mascot search engine (Matrix Science, London, UK, version: 2.4). The following search parameters were used; enzyme: trypsin, maximum missed cleavage sites: 0, precursor ion mass tolerance: 10 ppm, fragment ion tolerance: 0.05 Da, fixed modification: carbamidomethylation on cysteine, variable modifications: fucose addition on HexNAc residues, oxidation on methionine residues. For the bacterial O-glycosylation study, the letter O was assigned to diNAcBac residue in the unimod.xml file, as opposed to the HexNAc in N-glycosylation searches. In addition, mono- and di-acetylation was considered as variable modifications. All other parameters were similar as mentioned above.

### Identification of sialylated N-linked glycoproteins from serum samples

The raw LC-MS data sets from 24 serum samples were loaded to Mascot Distiller (Matrix Science, London, UK, version: 2.6.1.0) and corresponding peak lists in Mascot generic format (mgf) were generated using the peak processing and peak picking algorithms incorporated in Mascot Distiller. The peak lists were then searched against the custom N-linked glycoprotein database (406 glycoprotein entries) using Mascot search engine (Matrix Science, London, UK, version: 2.4). All the search parameters were the same as described above. Following the database search using Mascot’s standard protein grouping algorithm, glycopeptide identifications were only considered as positive hits with a Mascot ion score of 25, a top scoring match to a particular spectrum and a significance threshold *p-value* < 0.001.

### Relative quantification of sialylated N-linked glycoproteins from serum samples

Glycoproteins/peptides passing the above filtration criteria were imported and quantified relatively (aggressive vs indolent) using the replicate quantification protocol available in Mascot Distiller. Replicate protocol is a label free quantitation method based on extracted ion chromatogram intensities (XIC) of precursors in multiple samples, which are aligned using mass and elution time. The peptide peak areas were integrated using the Simpson’s method. An elution time window of 2 min was allowed to compensate the retention time shifts during the LC-MS runs. A lower limit of 0.8 for correlation coefficient between the predicted and observed precursor isotope distributions was used during the peak area calculations. Moreover, intensities of additional charge states must exceed the fraction (0.20) of most intense charge state to be considered for quantification. Apart from this, all other parameters were used default as suggested by Mascot Distiller. While calculating the peptide ratios (aggressive vs indolent), precursors which contribute to a minimum 50% of the XIC peak area and precursors passing the correlation threshold of 0.8 were only considered. The protein ratios are reported as median of the corresponding peptide ratios. The mass spectrometry proteomics data have been deposited to the ProteomeXchange Consortium^64^ via the PRIDE^65^ partner repository with the dataset identifier PXD005931.

## Electronic supplementary material

Supplementary Information

Supplementary Table 1

Supplementary Tables 2-5
