# Author Correction: Large-scale intact glycopeptide identification by Mascot database search

**DOI:** 10.1038/s41598-018-28147-w

**Published:** 2018-06-22

**Authors:** Ravi Chand Bollineni, Christian Jeffrey Koehler, Randi Elin Gislefoss, Jan Haug Anonsen, Bernd Thiede

**Affiliations:** 1 Department of Biosciences, University of Oslo, Oslo, Norway; 2 Cancer Registry of Norway, Institute of Populationbased Cancer Research, Oslo, Norway

Correction to: *Scientific Reports* 10.1038/s41598-018-20331-2, published online 01 February 2018

A supplementary file containing Supplementary Table 1 was omitted from the original version of this Article. This has been corrected in the HTML version of the Article; the PDF version was correct at time of publication.

