# Supplementary material for: Large-scale intact glycopeptide identification by Mascot database search
Source: Sci Rep. 2018 Feb 1;8:2117. doi: 10.1038/s41598-018-20331-2 (PMC5795011; doi:10.1038/s41598-018-20331-2)
Supplement: Supplementary file 1 — Supplementary Information [file 41598_2018_20331_MOESM1_ESM.pdf]

Supplementary figures and file

Large-scale intact glycopeptide identification by Mascot database search

Ravi Chand Bollineni[1], Christian Jeffrey Koehler[1], Randi Elin Gislefoss[2], Jan Haug Anonsen[1], and Bernd Thiede[1*]

[1]Department of Biosciences, University of Oslo, Oslo, Norway

[2]Cancer Registry of Norway, Institute of Population-based Cancer Research, Oslo, Norway

Supplementary Figure 1: Definitions of the three unique one letter codes for O (GlcNAc, GalNAc), J (Galactose, mannose) and U (Neu5Ac) as implemented in the unimod.xml file.

Supplementary Figure 2: Detailed description of the steps involved in the preparation of the custom glycoprotein database. An in-house written python script was used for preparation of the custom glycoprotein database and all these steps were performed automatically. Briefly, the proteins in the FASTA file were digested in silico assuming zero missed cleavages. Peptide sequences in between five and thirty amino acids were then scanned for NxS/T/C motifs (N-liked glycosylation) and the linear glycan sequences were attached to create a custom glycoprotein database.

Supplementary Figure 3: Mascot annotated HCD-MS2 spectra of glycopeptides from protein PilE containing diNAcBac (**Fig. 3A**), diNAcBac-Gal (**Fig. 3B**) residues. Mono- and di-acetylation is known to exist on these glycan residues. Mascot annotated both mono- (**Fig. 3C**) and di-acetylation (**Fig. 3D**) on galactose residues clearly defined by the b_2 ion at *m/z* 433.18 (**Fig. 3C**) and 475.19 (**Fig. 3D**). The diNAcBac residue was defined with letter O and galactose with letter J in unimod.xml file and acetylation was used as a variable modification.

Supplementary Figure 4: HCD-MS2 spectra of a tri-sialylated tri-antennary (**Fig. 3A**), di-sialylated tetra-antennary fucosylated peptides (**Fig. 3B**) of alpha-1-acid-glycoprotein 1 and di-sialylated bi-antennary fucosylated glycopeptide of alpha-2-macroglobulin (**Fig. 3C**). The inserts represent the presence or absence of a peak corresponding to the residue mass of fucose (+146 Da, +73 Da^{2+}) following the peptide+HexNAc peak.

Supplementary Figure 5: Violin plots showing the glycopeptide ratios (Aggressive vs indolent) of all the major glycan variants identified in the current study. Glycopeptide variants with at least 10 peptide ratio values were considered.

Supplementary Figure 6: Venn diagram showing the overlap of the glycoprotein identifications from Mascot search engine using different glycoprotein databases. The 24 LC-MS datasets from serum samples were searched against differentdatabases. 1) Plasma Glyco: All known plasma glycosylated proteins from PeptideAtlas N-Glyco 2010 (444 glycoproteins), 2) Plasma: all known plasma/serum proteins from PeptideAtlas build 2010 (2421 glycoproteins), 3) PNGaseF: all deamidated proteins identified following PNGaseF treatment of glycopeptides from the same 24 serum samples (280 glycoproteins) and 4) Human: Swiss-Prot annotated human proteome (14120 glycoproteins).

Supplementary Figure 7: HCD-MS2 spectra of asialo bi-antennary glycopeptides form Ig gamma-2 chain C region (**Fig. 6A, 6B**), and apolipoprotein B-100 (**Fig. 6C**). HCD-MS2 spectra of high mannose glycopeptide from complement C4-A is shown in **Fig. 6D**. The eluted peptides from the HILIC-SPE column from one of the serum samples was analyzed.

Supplementary File: The python script used for the preparation of glycoprotein databases.

Supplementary figure 1


```xml
  </umod:aa>
  <umod:aa avge_mass="203.1925" full_name="N-Acetyl_Hexosamine" mono_mass="203.079373" three_letter="HXN" title="O">
    <umod:element number="8" symbol="C"/>
    <umod:element number="13" symbol="H"/>
    <umod:element number="1" symbol="N"/>
    <umod:element number="5" symbol="O"/>
  </umod:aa>
  <umod:aa avge_mass="162.1406" full_name="Hexose" mono_mass="162.052823" three_letter="HEX" title="J">
    <umod:element number="10" symbol="H"/>
    <umod:element number="6" symbol="C"/>
    <umod:element number="5" symbol="O"/>
  </umod:aa>
  <umod:aa avge_mass="291.2550" full_name="N-Acetylneuraminic acid" mono_mass="291.095417" three_letter="Neu" title="U">
    <umod:element number="17" symbol="H"/>
    <umod:element number="11" symbol="C"/>
    <umod:element number="1" symbol="N"/>
    <umod:element number="8" symbol="O"/>
  </umod:aa>
</umod:amino_acids>
```

Supplementary figure 2

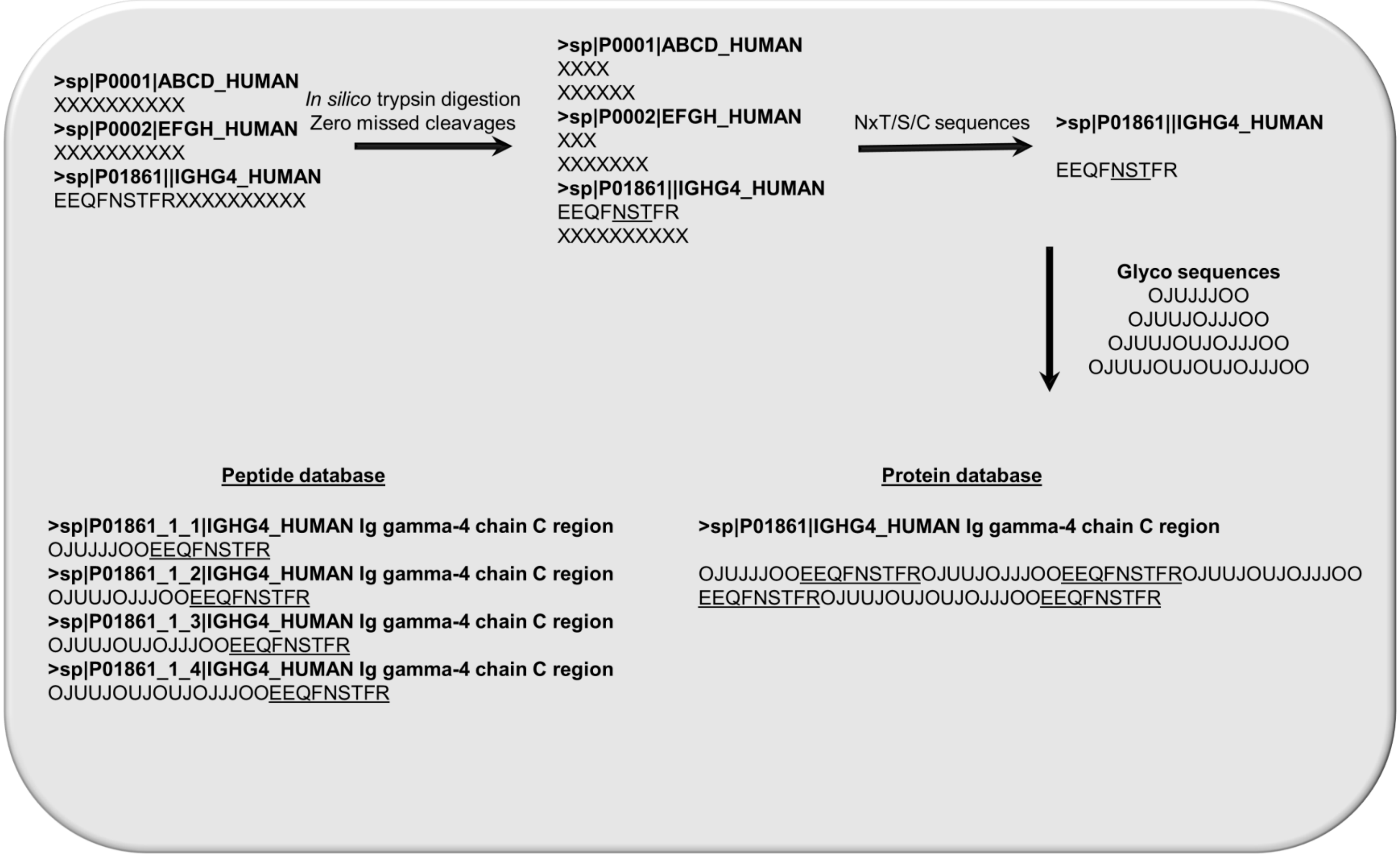

Supplementary figure 3

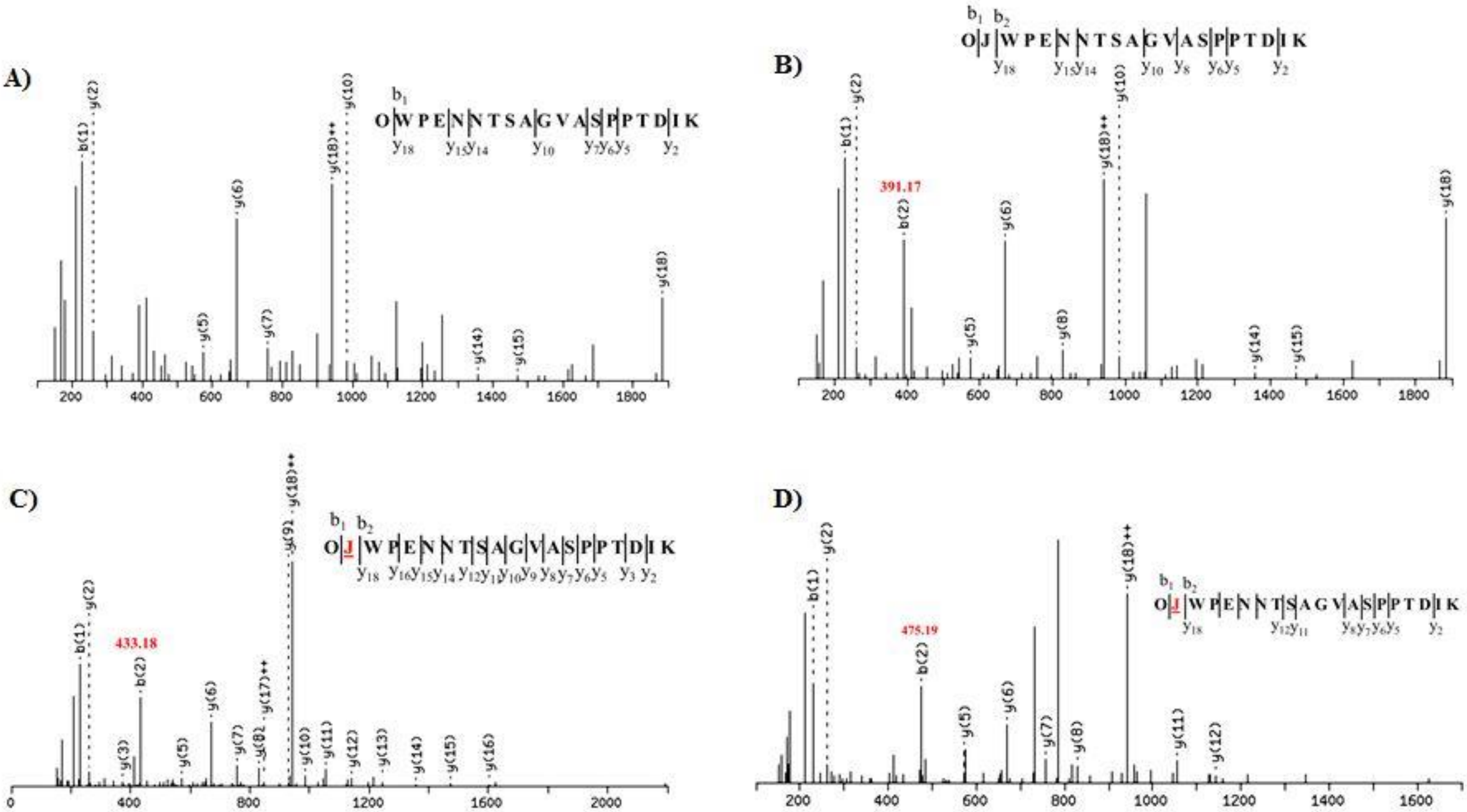

Supplementary figure 4

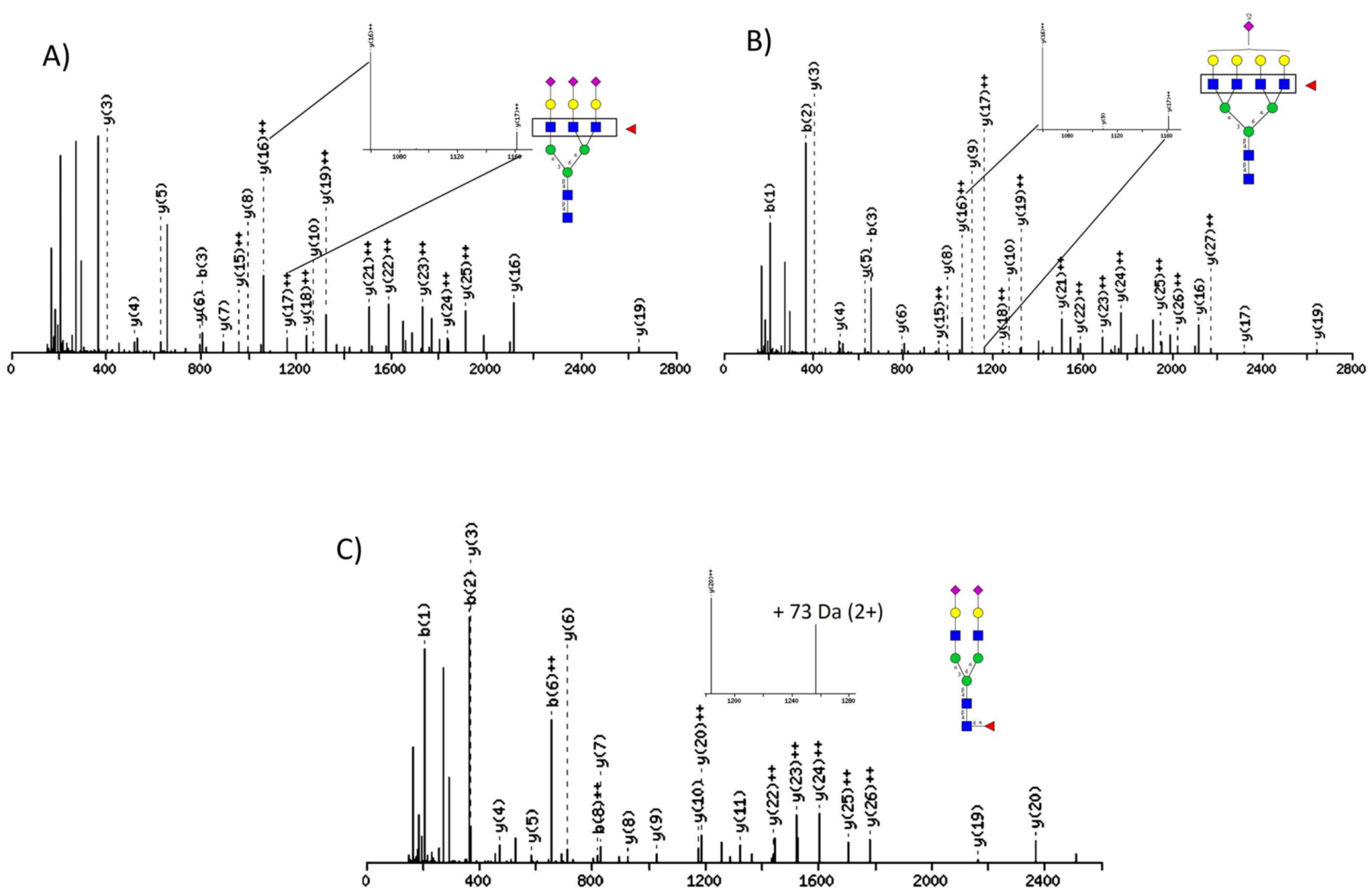

Supplementary figure 5

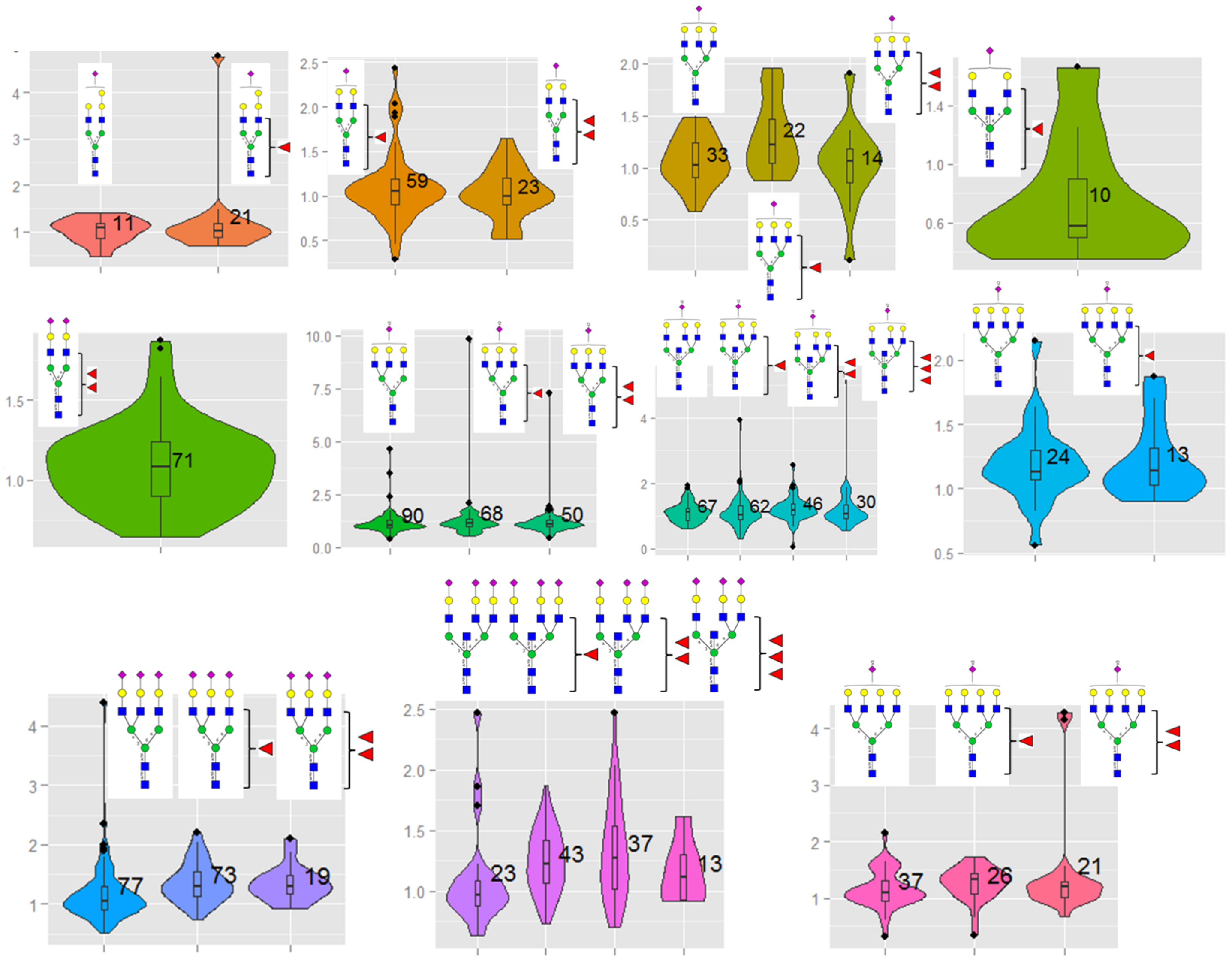

Supplementary figure 6

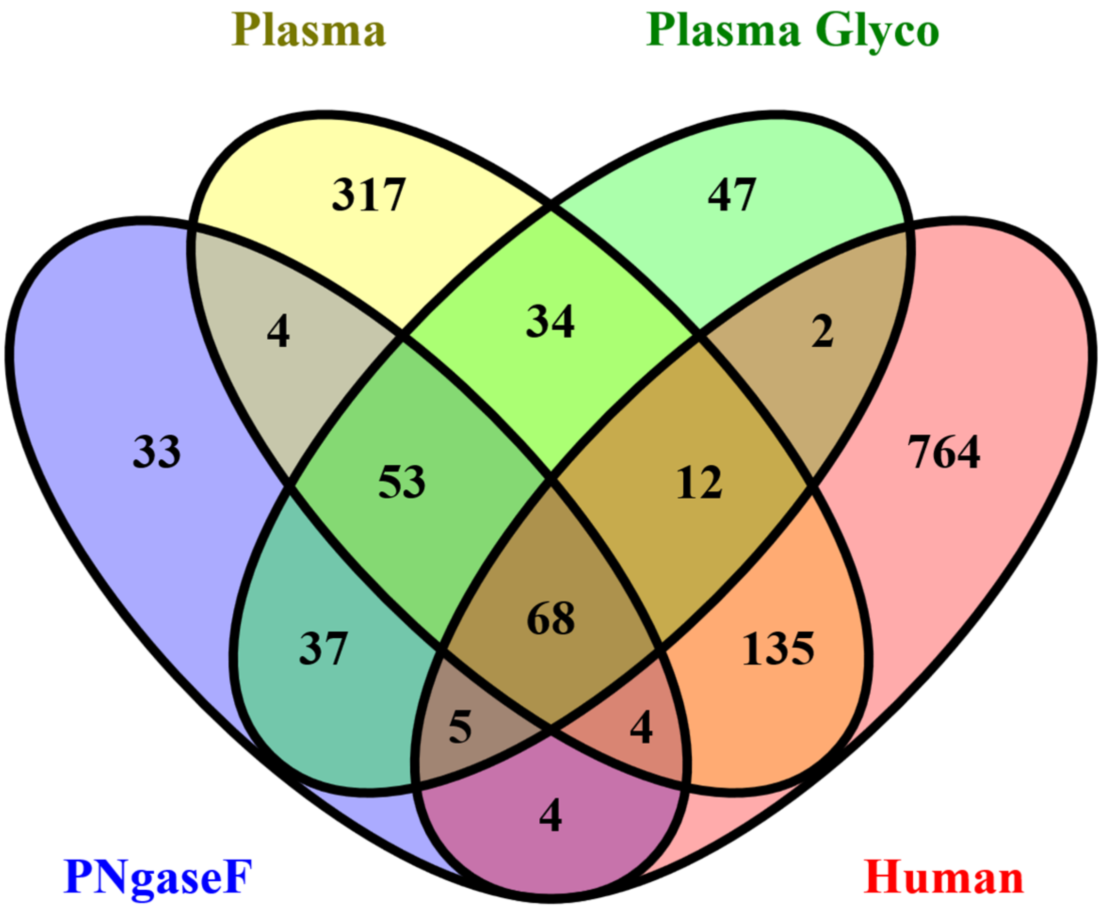

Supplementary figure 7

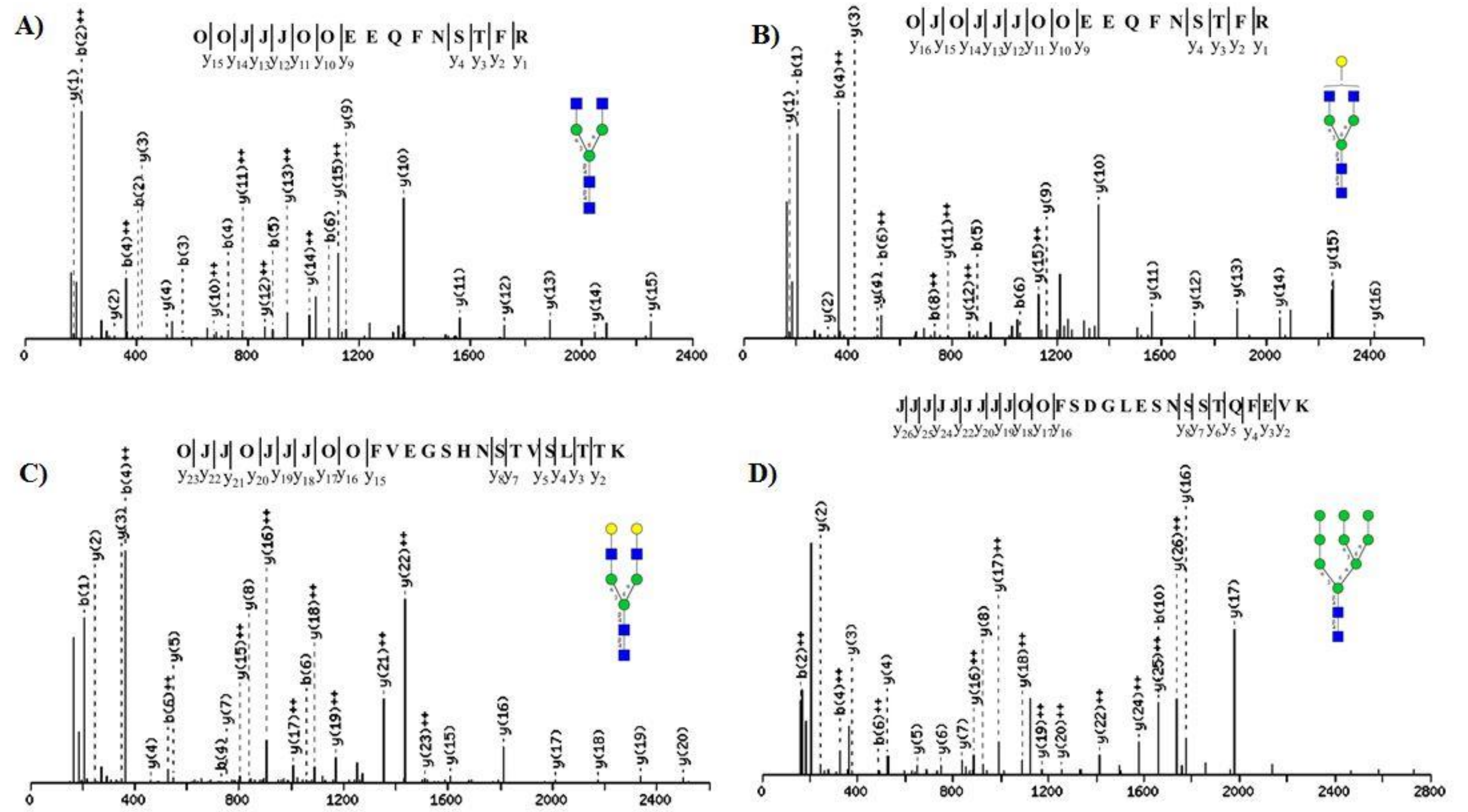

Supplementary file

```python
import os
import sys
import re
from pyteomics import fasta, parser

def transformFasta(fastafile,glycofile):

    #read the fils to marry
    glycos = read_glyco(glycofile)
    proteins = list(fasta.read(fastafile))

    #open new fastafiles
    proteinlevel_fasta = open('Glyco_prot_%s' %fastafile,'w')
    peptidelevel_fasta = open('Glyco_pep_%s' %fastafile,'w')
    glycolevel_fasta = open('Glyco_glyco_%s' %fastafile,'w')


    #counters
    procounter = 0
    pepcounter = 0



    for protein in proteins:
        proteinlevel_sequences = ''
        pep_appendix = 1

        prot, seq = protein
        acc,ident,descr = splitter(prot)

        procounter +=1
        if procounter%1000==0:
                print '---->  Process protein %s:%s' %(procounter,ident)

        peptides = parser.cleave(seq,parser.expasy_rules['trypsin'],\
                                 missed_cleavages=0, min_length=5)

        for pep in peptides:
      if len(pep) < 30:
        peptidelevel_sequences = ''
        glyco_appendix = 1

        pepcounter+=1
        if pepcounter%10000==0:
          print 'Process peptide %s' %pepcounter

        if re.search('N.[TSC]', pep):  # find the pattern NxT, NxS or NxC in the peptide
          #peptides_motiv.append(pep)
          for glyco in glycos:

            #adding the peptides to the sequences
            proteinlevel_sequences += '%s%s' %(glyco,pep)
            peptidelevel_sequences += '%s%s' %(glyco,pep)
            #for the glycolevel sequence, we can write it right away
            glycolevel_fasta.write('>sp|%s_%s_%s|%s %s\n' \
              %(acc,str(pep_appendix),str(glyco_appendix),ident,descr))
            glycolevel_fasta.write('%s%s\n' %(glyco,pep))
            glyco_appendix +=1

          #for the peptidelevel_sequences we write the fasta
          peptidelevel_sequences = insert_newlines(
```

```
peptidelevel_sequences)
          peptidelevel_fasta.write('>sp|%s_%s|%s %s\n' %(acc,str(pep_appendix),ident,descr)),
          peptidelevel_fasta.write('%s\n' %(peptidelevel_sequences))
          pep_appendix +=1

        if proteinlevel_sequences:
            #for the proteinlevel_sequences we write the fasta
            proteinlevel_sequences = insert_newlines(proteinlevel_sequences)
            proteinlevel_fasta.write('>sp|%s|%s %s\n' %(acc,ident,descr))
            proteinlevel_fasta.write('%s\n' %(proteinlevel_sequences))

    #closing the new files
    glycolevel_fasta.close()
    peptidelevel_fasta.close()
    proteinlevel_fasta.close()
    print 'Done'


def read_glyco(glycofile):
    ''' reads a .txt file with glyco sequences and returns a list of glyco sequences'''
    glycos = open(glycofile,'r').readlines()
    return [i.strip() for i in glycos]

def insert_newlines(string, every=64):
    return '\n'.join(string[i:i+every] for i in xrange(0, len(string), every))

def splitter(p):
    start = p.find('|')
    middle = p.find('|',start+1)
    middle2 = p.find(' ',middle+1)
    end = p.find('OS=')
    return p[start+1:middle],p[middle+1:middle2], p[middle2+1:end].strip()



if __name__ == '__main__':

    if len(sys.argv) <3:
        print '\n***************************************************\n\
Use as follows:'
        print '"Generate_Glycodb.py fastafile.fasta glycofile.txt"\
        \n***************************************************\n'
    else:
        transformFasta(sys.argv[1],sys.argv[2])
```

```
import os
import sys
import re
from pyteomics import fasta, parser

def transformFasta(fastafile,glycofile):

    #read the fils to marry
    glycos = read_glyco(glycofile)
    proteins = list(fasta.read(fastafile))

    #open new fastafiles
    proteinlevel_fasta = open('Glyco_prot_%s' %fastafile,'w')
    peptidelevel_fasta = open('Glyco_pep_%s' %fastafile,'w')
    glycolevel_fasta = open('Glyco_glyco_%s' %fastafile,'w')


    #counters
    procounter = 0
    pepcounter = 0



    for protein in proteins:
        proteinlevel_sequences = ''
        pep_appendix = 1

        prot, seq = protein
        acc,ident,descr = splitter(prot)

        procounter +=1
        if procounter%1000==0:
                print '---->  Process protein %s:%s' %(procounter,ident)

        peptides = parser.cleave(seq,parser.expasy_rules['trypsin'],\
                                 missed_cleavages=0, min_length=5)

        for pep in peptides:
            if len(pep) < 30:
                peptidelevel_sequences = ''
                glyco_appendix = 1

                pepcounter+=1
                if pepcounter%10000==0:
                    print 'Process peptide %s' %pepcounter

                #if re.search('N.[TSC]', pep):  # find the pattern NxT, NxS or NxC in the peptide
                if re.search('[TS]', pep):  # find Threonin or Serin in the peptide
                    #peptides_motiv.append(pep)
                    for glyco in glycos:

                        #adding the peptides to the sequences
                        proteinlevel_sequences += '%s%s' %(glyco,pep)
                        peptidelevel_sequences += '%s%s' %(glyco,pep)
                        #for the glycolevel sequence, we can write it right away
                        glycolevel_fasta.write('>sp|%s_%s_%s|%s %s\n' \
                            %(acc,str(pep_appendix),str(glyco_appendix),ident,descr))
                        glycolevel_fasta.write('%s%s\n' %(glyco,pep))
```

```
                        glyco_appendix +=1

                    #for the peptidelevel_sequences we write the fasta
                    peptidelevel_sequences = insert_newlines(peptidelevel_sequences)
                    peptidelevel_fasta.write('>sp|%s_%s|%s %s\n' %(acc,str(pep_appendix),ident,descr)),
                    peptidelevel_fasta.write('%s\n' %(peptidelevel_sequences))
                    pep_appendix +=1

        if proteinlevel_sequences:
            #for the proteinlevel_sequences we write the fasta
            proteinlevel_sequences = insert_newlines(proteinlevel_sequences)
            proteinlevel_fasta.write('>sp|%s|%s %s\n' %(acc,ident,descr))
            proteinlevel_fasta.write('%s\n' %(proteinlevel_sequences))

    #closing the new files
    glycolevel_fasta.close()
    peptidelevel_fasta.close()
    proteinlevel_fasta.close()
    print 'Done'


def read_glyco(glycofile):
    ''' reads a .txt file with glyco sequences and returns a list of glyco sequences'''
    glycos = open(glycofile,'r').readlines()
    return [i.strip() for i in glycos]

def insert_newlines(string, every=64):
    return '\n'.join(string[i:i+every] for i in xrange(0, len(string), every))

def splitter(p):
    start = p.find('|')
    middle = p.find('|',start+1)
    middle2 = p.find(' ',middle+1)
    end = p.find('OS=')
    return p[start+1:middle],p[middle+1:middle2], p[middle2+1:end].strip()



if __name__ == '__main__':

    if len(sys.argv) <3:
        print '\n***************************************************\n\
Use as follows:'
        print '"Generate_Glycodb.py fastafile.fasta glycofile.txt"\
        \n***************************************************\n'
    else:
        transformFasta(sys.argv[1],sys.argv[2])
```
