# Supplementary material for: Large-scale intact glycopeptide identification by Mascot database search
Source: Sci Rep. 2018 Feb 1;8:2117. doi: 10.1038/s41598-018-20331-2 (PMC5795011; doi:10.1038/s41598-018-20331-2)
Supplement: Supplementary file 2 — Supplementary Table 1 [file 41598_2018_20331_MOESM2_ESM.doc]

**Supplementary table 1:** List of major glycan structures and proposed linear sequences.

|  | Glycan structure | Linear Sequence | Monoisotopic Mass | Peptide bound  Residue mass |
| --- | --- | --- | --- | --- |
| 1 | 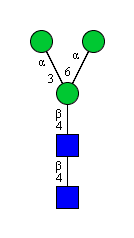 | JJJOO | 910.32778 | 892.31722 |
| 2 | 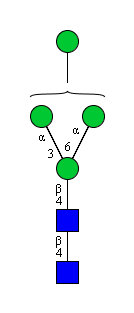 | JJJJOO | 1072.3806 | 1054.3700 |
| 3 | 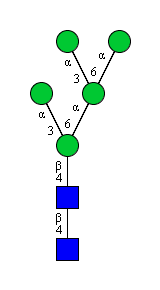 | JJJJJOO | 1234.4333 | 1216.4228 |
| 4 | 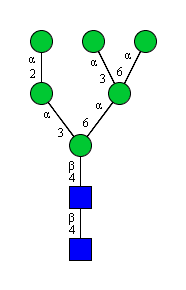 | JJJJJJOO | 1396.4862 | 1378.4756 |
| 5 | 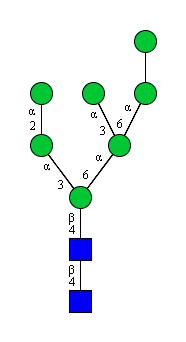 | JJJJJJJOO | 1558.5390 | 1540.5285 |
| 6 | 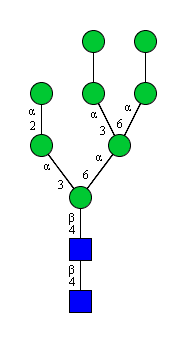 | JJJJJJJJOO | 1720.5919 | 1702.5813 |
| 7 | 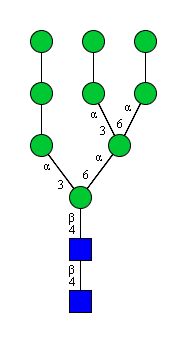 | JJJJJJJJJOO | 1882.6447 | 1864.6341 |
| 8 | 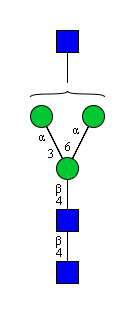 | OJJJOO | 1113.4071 | 1095.3965 |
| 9 | 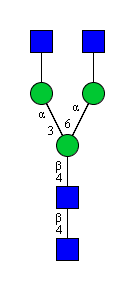 | OOJJJOO | 1316.4865 | 1298.4759 |
| 10 | 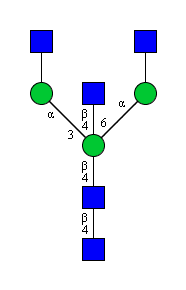 | OOJJOJOO | 1519.5659 | 1501.5553 |
| 11 | 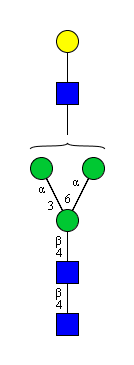 | OJJJJOO | 1275.4599 | 1257.4494 |
| 12 | 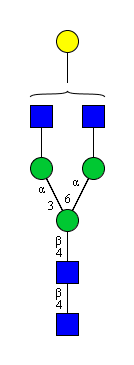 | OJOJJJOO | 1478.5393 | 1460.5287 |
| 13 | 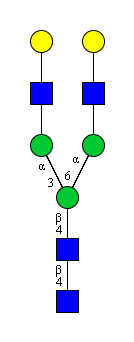 | OJJOJJJOO | 1640.5921 | 1622.5816 |
| 14 | 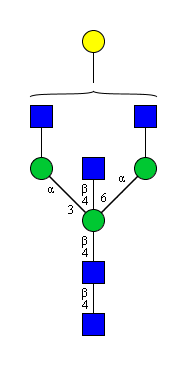 | OJOOJJJOO | 1681.6187 | 1663.6081 |
| 15 | 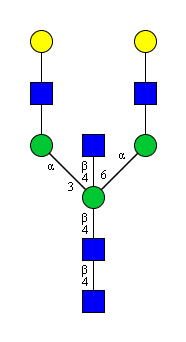 | OJOJOJJJOO | 1843.6715 | 1825.6609 |
| 16 | 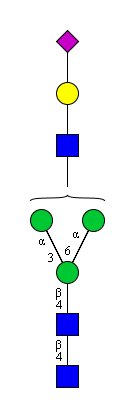 | OJUJJJOO | 1566.5553 | 1548.5448 |
| 17 | 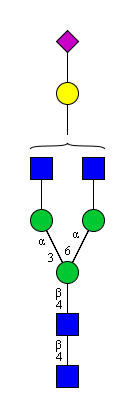 | OJUOJJJOO | 1769.6347 | 1751.6242 |
| 18 | 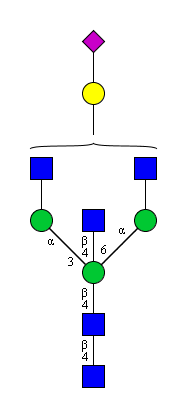 | OJUOOJJJOO | 1972.7141 | 1954.7035 |
| 19 | 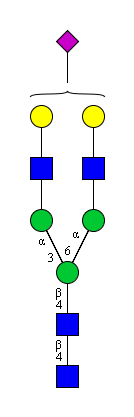 | OJUJOJJJOO | 1931.6875 | 1913.6770 |
| 20 | 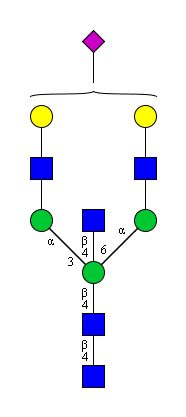 | OJUOJOJJJOO | 2134.7669 | 2116.7564 |
| 21 | 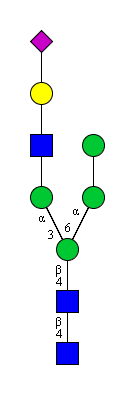 | OJUJJJJOO | 1728.6082 | 1710.5976 |
| 22 | 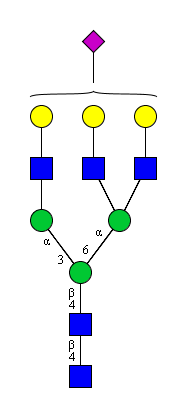 | OJUJOJOJJJOO | 2296.8197 | 2278.8092 |
| 23 | 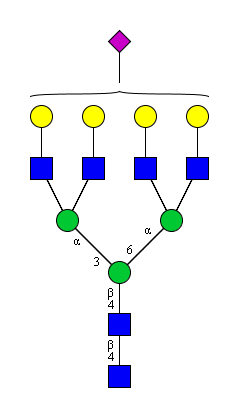 | OJUJOJOJOJJJOO | 2661.9519 | 2643.9414 |
| 24 | 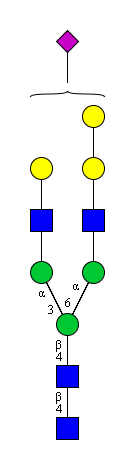 | OJUJJOJJJOO | 2093.7404 | 2075.7298 |
| 25 | 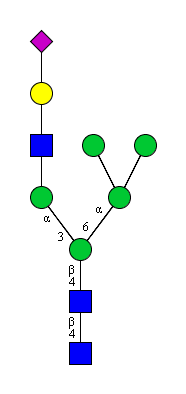 | OJUJJJJJOO | 1890.6601 | 1872.6504 |
| 26 | 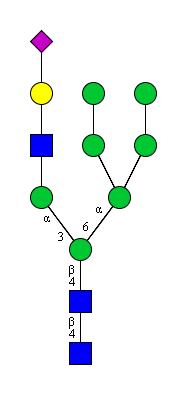 | OJUJJJJJJJOO | 2214.7666 | 2196.7561 |
| 27 | 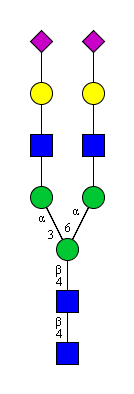 | OJUUJOJJJOO | 2222.7830 | 2204.7724 |
| 28 | 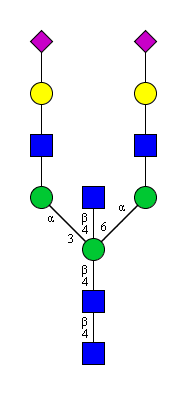 | OJUOUJOJJJOO | 2425.8623 | 2407.8518 |
| 29 | 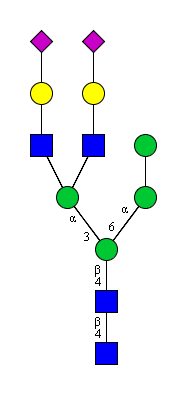 | OJUJUJOJJJOO | 2384.8358 | 2366.8252 |
| 30 | 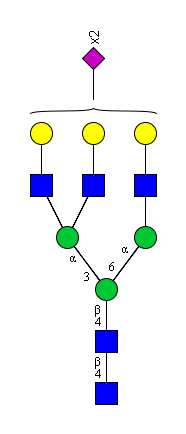 | OJUUJOJOJJJOO | 2587.9152 | 2569.9046 |
| 31 | 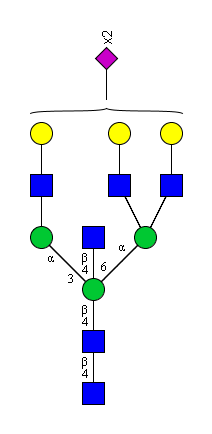 | OJUUJOJOJJOJOO | 2790.9945 | 2772.9840 |
| 32 | 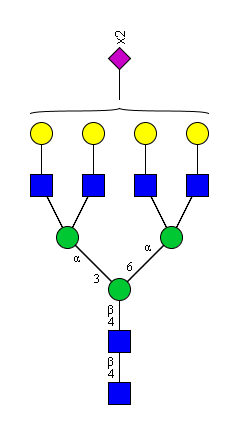 | OJUUJOJOJOJJJOO | 2953.0474 | 2935.0368 |
| 33 | 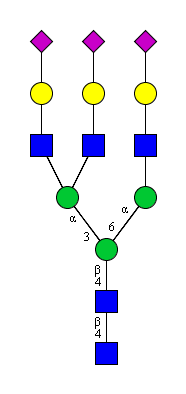 | OJUUJOUJOJJJOO | 2879.0106 | 2861.0000 |
| 34 | 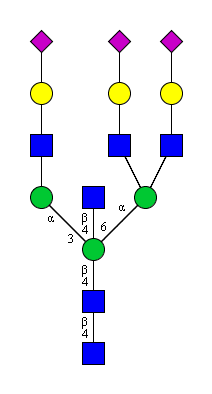 | OJUUJOUJOJJOJOO | 3082.0899 | 3064.0794 |
| 35 | 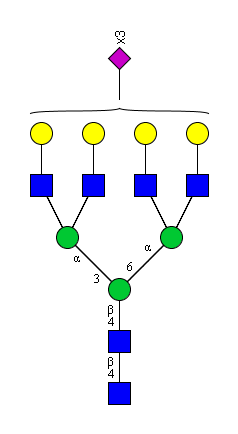 | OJUUJOUJOJOJJJOO | 3244.14281 | 3226.1322 |
| 36 | 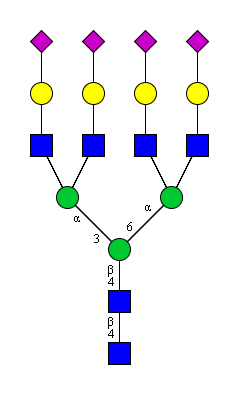 | OJUUJOUJOUJOJJJOO | 3535.2382 | 3517.2276 |
| 37 | 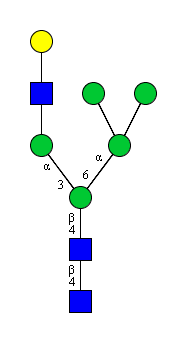 | OJJJJJJOO | 1599.5656 | 1581.5550 |
| 38 | 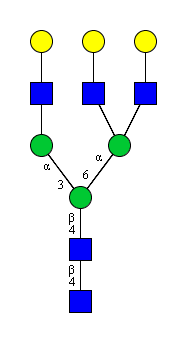 | OJJOJOJJJOO | 2005.7243 | 1987.7138 |
| 39 | 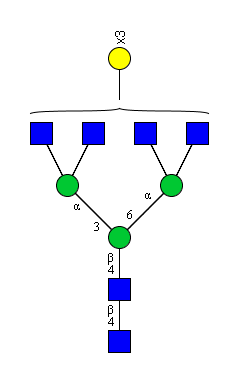 | OJOJOJOJJJOO | 2208.8037 | 2190.7931 |
| 40 | 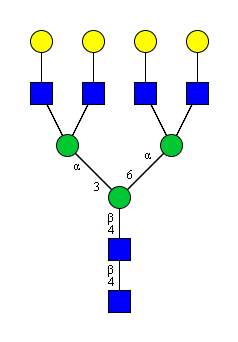 | OJJOJOJOJJJOO | 2370.8568 | 2352.8460 |
